# Neuronal FGF13 Inhibits Mitochondria‐Derived Damage Signals to Prevent Neuroinflammation and Neurodegeneration in a Mouse Model of Parkinson's Disease

**DOI:** 10.1002/advs.202503579

**Published:** 2025-05-08

**Authors:** Nanshan Song, Xiangxu Wang, Luqing Ha, Lamei Hu, Shuyuan Mei, Yue Liang, Yujie Zhao, Xingyin Yang, Qingyu Zhang, Yuanzhang Zhou, Jianhua Ding, Yan Liu, Qigang Zhou, Feng Han, Gang Hu, Ming Lu

**Affiliations:** ^1^ School of Medicine Nanjing University of Chinese Medicine Nanjing 210023 China; ^2^ Jiangsu Key Laboratory of Neurodegeneration Department of Pharmacology Nanjing Medical University Nanjing 211166 China; ^3^ School of Pharmacy Nanjing University of Chinese Medicine Nanjing 210023 China; ^4^ The Clinical Medical College Nanjing Medical University Nanjing 211166 China; ^5^ Changzhou Second People's Hospital Changzhou Medical Center Nanjing Medical University Changzhou 213000 China

**Keywords:** FGF13, mitochondrial transfer, MTCH2, neuroinflammation, parkinson's disease

## Abstract

Fibroblast growth factor homologous factors (FHFs) are highly expressed in the central nervous system (CNS). It is demonstrated that the FHFs subfamily plays cardinal roles in several neuropathological diseases, while their involvement in Parkinson's disease (PD) has been so far scarcely investigated. From the publicly available Gene Expression Omnibus (GEO) datasets, *FHF2* (also known as fibroblast growth factor 13, *FGF13*) alterations are described in PD patients. *Fgf13* gene is significantly decreased in several PD mouse models, and its overexpression alleviates the PD‐like pathological phenotype. Although FGF13 is highly expressed in neurons, it functions by preventing glia‐dependent inflammatory processes. Mechanistically, FGF13 combines mitochondrial proteins such as MCHT2 (a protein localized on the mitochondrial outer membrane), to anchor mitochondria within the cytoplasm. Under PD‐related stress, decreased neuronal FGF13 levels induce the release of the damaged mitochondria, which in turn activate microglia and astrocytes, thereby promoting neurodegeneration. Abacavir, an FDA‐applied anti‐retroviral drug, is identified to prevent excessive gliosis and neuron loss in both glia‐neuron co‐cultures and PD mouse models by rejuvenating FGF13 signaling. Collectively, neuronal FGF13 inhibits the transfer of stressed mitochondria to glia, thereby impeding neuroinflammation and neurodegeneration. Abacavir is a promising neuroprotectant and sets a brake to PD progression.

## Graphical abstract

This study elucidates a novel role of FGF13 in manipulating neuronal fate via mitochondrial transfer. FGF13 is identified as a mitochondria‐stabilizing protein by interacting with mitochondrial proteins. Under stress, the decrease of neuronal FGF13 fails to retain mitochondria within the cytoplasm, leading to the release of damaged mitochondria to trigger glial reactivity and propagate neurodegeneration.

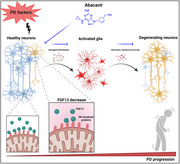

## 1 Introduction

Parkinson's disease (PD) is a neurodegenerative disorder characterized by progressive dopaminergic neuron loss in the substantia nigra pars compacta (SNpc) of the midbrain and a substantial decline of dopamine levels in the striatum. These disruptions within the nigrostriatal dopaminergic system lead to a spectrum of motor symptoms, typically manifested as postural instability and loss of motor control.^[^ ^1^ ^]^ Currently, the clinically‐used therapies for PD primarily provide symptomatic relief of motor symptoms but fail to delay disease progression.^[^ ^2^ ^]^ This incurable situation of PD, to a significant extent, is attributed to imprecise understanding of the disease pathogenesis.^[^ ^1^ ^]^ In recent years, the application of omics approaches to PD patients’ samples has continuously updated our understanding of key targets and critical biological processes that contribute to neuronal susceptibility in PD. Thereupon, we aim to explore novel targets for PD by comprehensively analyzing RNA sequencing (RNA‐seq) data derived from the substantia nigra (SN) and striatum of PD patients in the Gene Expression Omnibus (GEO) public datasets.

Neurons, as the basic working unit of the brain, and their activities directly influence the physiological and pathological states of the brain.^[^ ^3^ ^]^ Glial cells (mainly astrocytes, microglia, and oligodendrocytes) are numerically dominant.^[^ ^4^ ^]^ These cells actively support the neuronal functions,^[^ ^4^ ^]^ and also act as indispensable participants of neuronal damage under pathological conditions.^[^ ^5^, ^6^ ^]^ It has been reported that pathological alpha‐synuclein (α‐syn) fibrills induce microglia to facilitate α‐syn transmission between the recipient neurons.^[^ ^7^ ^]^ The neurotoxic astrocytes induced by disease‐related microglia also propagate neurodegeneration to a greater extent.^[^ ^8^ ^]^ Conversely, neurons release waste materials to adjacent astrocytes for disposal and degradation.^[^ ^9^ ^]^ Therefore, the neuron‐glia interplay plays a core role in maintaining brain physiology and boosting neurological pathology.

Mitochondria, often regarded as the energy factories of cells, have recently been emerged as critical mediators for intercellular communication.^[^ ^10^ ^]^ Both the contents of mitochondria including various enzymes, metabolic substrates and products, as well as the mitochondria themselves are constantly transferred and exchanged between brain cells.^[^ ^10^ ^]^ Studies have shown that neurons can release damaged mitochondria to neighboring astrocytes for recycling,^[^ ^9^ ^]^ and astrocytes reversely transfer functional mitochondria to rescue stressed neurons.^[^ ^11^ ^]^ Several groups of nerve cells have been reported to communicate through the mitochondrial transfer,^[^ ^10^ ^]^ highlighting that mitochondria‐mediated cell crosstalk represents critical nodes within neural networks under both physiological and pathological conditions.

Fibroblast growth factors (FGFs) constitute a large family of structurally related proteins, which can be classified into secretory forms (FGF1‐10 and FGF15‐23) and non‐secretory forms (FGF11‐14).^[^ ^12^ ^]^ The non‐secretory FGFs, also known as FGF homologous factors (FHFs), share similarities in gene sequences and structures with other FGF family members, but exhibit higher expression in the central nervous system (CNS), particularly in neurons.^[^ ^13^ ^]^ FHFs primarily exert their functions intracellularly by interacting with various proteins, including IB2, voltage‐gated sodium channels (VGSCs), and tubulin, to modulate neuronal excitability, polarization, and neurogenesis.^[^ ^14^ ^]^ Although FHFs lack conventional signal peptides for secretion, recent studies have demonstrated that certain FHFs can be secreted into the extracellular space through unconventional mechanisms,^[^ ^15^, ^16^ ^]^ thereby interacting with membranous FGF receptors. Gene mutations in FHFs often lead to neurological diseases.^[^ ^17^, ^18^, ^19^ ^]^ However, the involvement of FHF family members in PD has been so far scarcely investigated. Given the crucial roles of FHF family members in neuronal functions, we hypothesize that FHFs may play a significant role in manipulating the fate of dopaminergic neurons under stress.

In this study, we conducted a comprehensive analysis of RNA‐seq data derived from PD patient samples available in the GEO public datasets, followed by a series of experimental investigations. Our findings revealed that FGF13 expression was significantly decreased in the nigrostriatal pathway of both PD patients and mouse models. *Fgf13* overexpression was sufficient to drastically reverse dopaminergic degeneration and motor impairments in vivo, while it prevented neuronal damage only in the presence of glial cells in vitro. Mechanistically, FGF13 combined multiple mitochondrial proteins, such as MCHT2 of the mitochondrial outer membrane, to retain the mitochondria within the cytoplasm. Under stressful conditions, decreased FGF13 levels lead to the release of mildly damaged mitochondria, which in turn activate glial cells and promote neurodegeneration. Finally, we showed that Abacavir was repurposed to protect neurons from degeneration by increasing FGF13 levels. Therefore, the genetic or pharmacological up‐regulation of FGF13 represents a novel therapeutic approach for preventing mitochondria‐derived damage signals. Our study underscores Abacavir as a promising candidate for the treatment of PD.

## 2 Results

### 2.1 FGF13 is Decreased in Nigrostriatal System of Parkinson's Disease Patients and Parkinsonian Mice

**Figure 1:**
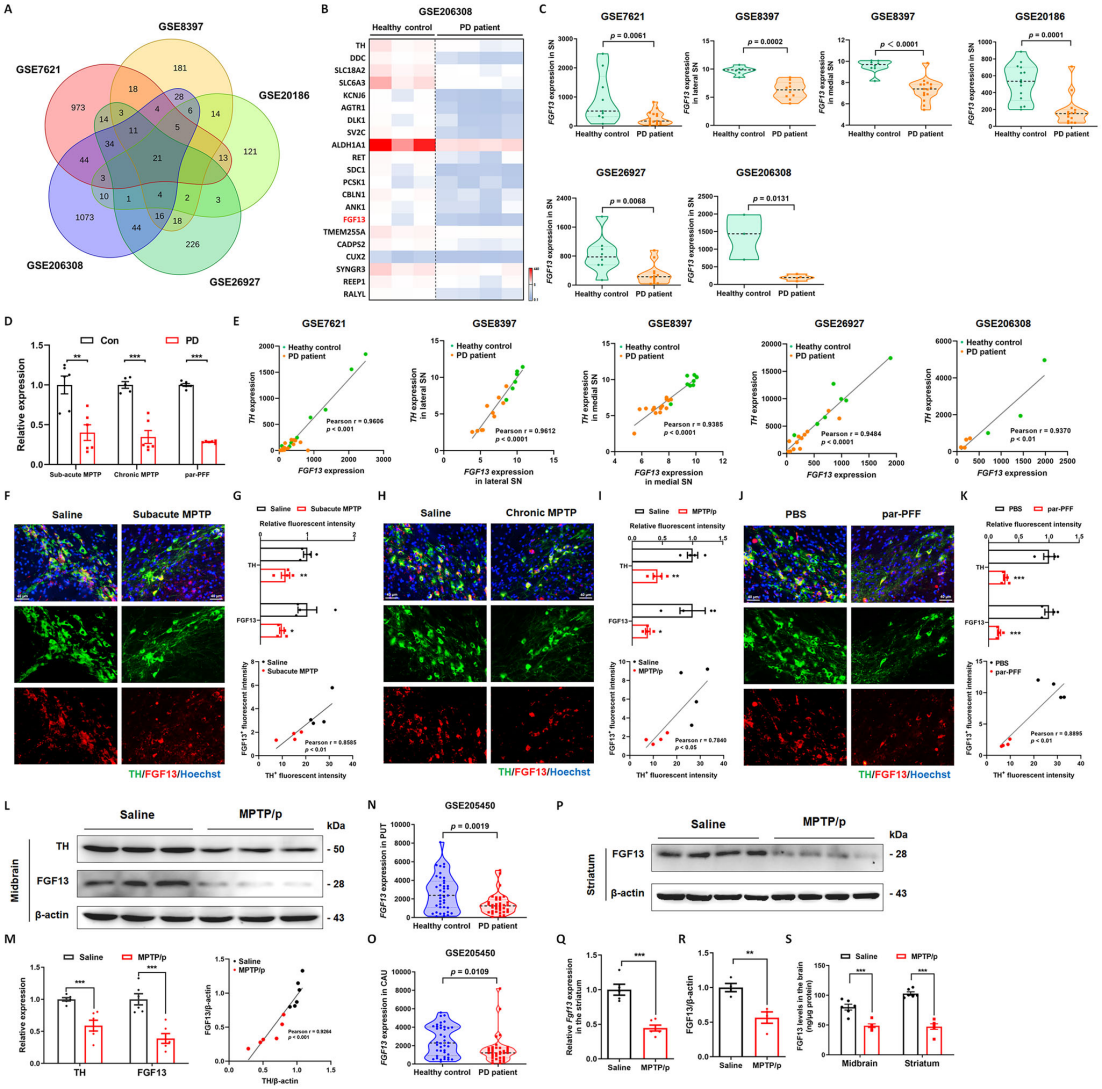
FGF13 is decreased in the nigrostriatal system of Parkinson's disease patients and parkinsonian mice. A). Venn diagram depicting the overlapped DEGs (Fold change > 2; P value < 0.05) in published RNA‐seq datasets (GSE7621, GSE8397, GSE20186, GSE26927 and GSE206308). B). Heat map of the overlapping DEGs expression in GES206308 (*n*  =  3 for the healthy group, *n*  =  4 for the disease group). C). *FGF13* gene expression in the SN of PD patients from the published RNA‐seq datasets (GSE7621: *n*  =  9 for the healthy group, *n*  =  16 for the disease group; GSE8397‐GPL96 platform: *n*  =  6 for the lateral SN of healthy group, *n*  =  10 for the lateral SN of disease group; GSE8397‐GPL96 platform: *n*  =  8 for the medial SN of healthy group, *n*  =  15 for the medial SN of disease group; GSE20186‐GPL96 platform: *n*  =  14 for the healthy group, *n* = 14 for the disease group; GSE26927: *n*  =  8 for the healthy group, *n* = 12 for the disease group; GSE206308: *n*  =  3 for the healthy group, n  =  4 for the disease group). D). *Fgf13* mRNA level in the midbrain of PD mouse models (*n* = 5–6 mice per group). E). The correlation between *FGF13* and *TH* gene expression in the SN of PD patients from the published RNA‐seq datasets. F). Representative fluorescent images showing FGF13 (red) and TH (green) in the SNc of mice subjected to subacute MPTP administration. G). The relative fluorescent intensities of TH and FGF13 in the SNc of mice subjected to subacute MPTP administration, along with the correlation between them (*n* = 4 mice per group). H). Representative fluorescent images demonstrating FGF13 (red) and TH (green) in the SNc of mice under chronic MPTP administration (MPTP/p mouse model). I). The relative fluorescent intensities of TH and FGF13 in the SNc of MPTP/p mice, as well as the correlation between them (*n* = 4 mice per group). J). Representative fluorescent images depicting FGF13 (red) and TH (green) in the SNc of mice with α‐synuclein PFF micro‐injection. K). The relative fluorescent intensity of TH and FGF13 in the SNc mice with α‐synuclein PFF micro‐injection, along with the correlation between them (*n* = 4 mice per group). L). Representative immunoblots of TH and FGF13 in the midbrain of MPTP/p‐administrated mice. M). Quantitative analysis of TH and FGF13 in the midbrain of MPTP/p‐administrated mice, and the correlation (*n* = 6 per group). N). *FGF13* gene expression in the putamen (PUT) of the PD patients from published RNA‐seq datasets (GSE205450: *n*  =  40 for the PUT of healthy group, *n*  =  35 for the PUT of disease group). **O**. *FGF13* gene expression in the caudate (CAU) of the PD patients from published RNA‐seq datasets (GSE205450: *n*  =  40 for the CAU of healthy group, *n * =  35 for the CAU of disease group). P). Representative immunoblots of FGF13 in the striatum of PD mice subjected to chronic MPTP administration. Q). *Fgf13* mRNA level in the striatum of MPTP/p mice (*n* = 5–6 mice per group). R). Quantitative analysis of FGF13 levels in the striatum of MPTP/p mice (*n* = 4 mice per group). S). FGF13 levels in tissue lysates from the midbrain and striatum of MPTP/p mice measured using an ELISA assay (*n* = 6 mice per group). All data are presented as the mean ± s.e.m. ^*^ *p* < 0.05, ^**^ *p* < 0.01, ^***^ *p* < 0.001. In C; D; the upper panel of G, I, and K; left panel of M; N; O; Q; R, and S, an unpaired two‐tailed Student's t‐test was used. In E; lower panel of G, I, and K; right panel of M, a Pearson correlation test was used.

Mechanisms underlying the neuronal susceptibility of nigrostriatal dopaminergic pathway in PD remain elusive. We therefore systematically analyzed the publicly available sequencing data derived from the SN tissues of PD patients in the GEO datasets. A summary of the datasets was provided in Figure S1A (Supporting Information). Our analysis identified 21 overlapping differentially expressed genes (DEGs) that were significantly changed in the SN of PD patients across several datasets (GSE7621, GSE8397, GSE20186, GSE26927, and GSE206308). Notably, *FGF13*, along with genes specifically expressed on dopaminergic neurons such as *TH*, *DDC*, *SLC18A2*, and others, showed a marked decrease (**Figure** **1**A–C). Subsequently, tissues from mouse models for PD were collected to verify the alterations of FGF13 expression across different PD models. As shown in Figure 1D, *Fgf13* mRNA levels were consistently decreased in the midbrain of these PD mouse models by quantitative real‐time polymerase chain reaction (qRT‐PCR) analysis. Intriguingly, in most of the patient‐derived GEO datasets, a positive correlation was observed between the *FGF13* gene level and the *TH* level (Figure 1E). However, in multiple datasets, including GSE49036 and GSE114517, *FGF13* expression was significantly reduced, while *TH* expression remained relatively unchanged (Figure S1B,C, Supporting Information). Additionally, *FGF13* levels exhibited a decreasing trend in datasets such as GSE20333, GSE42966, GSE136666, and GSE168496, although this decrease was not statistically significant (Figure S1D–G, Supporting Information). When considering all these datasets comprehensively, the *FGF13* gene level was positively correlated with the *TH* gene level (Figure S1B–G, Supporting Information). This correlation strongly indicates that the decline in *FGF13* in PD patients is synchronized with the loss of dopaminergic neurons. We also profiled gene changes for other FGF family members in the GEO databases of PD patients and found that FGF13 was the only gene that consistently showed a decrease across all datasets analyzed (Figure S1H–M, Supporting Information). In PD mouse models, we revealed that FGF13‐positive particles were localized within TH‐positive dopaminergic neurons, and FGF13 signals were significantly reduced in the midbrain of both 1‐methyl‐4‐phenyl‐1,2,3,6‐tetrahydropyridine (MPTP)‐treated and par‐α‐synuclein preformed fibrils (par‐PFF)‐treated mice (Figure 1F,H,J). The immunofluorescent signals of FGF13 and TH were synchronously down‐regulated with a positive correlation (Figure 1G,I,K). Meanwhile, levels of FGF13 protein were decreased (Figure 1L,M,S), and showed a positive correlation with TH protein in the midbrain of chronic MPTP‐treated mice (Figure 1L,M). We also retrieved the sequencing data from the striatum of Parkinsonian patients in the GEO datasets. It was demonstrated *FGF13* gene level was significantly decreased in patients’ putamen and caudate (Figure 1N,O). *Fgf13* mRNA and FGF13 protein were also decreased in the striatum of chronic MPTP‐treated mice by immunoblotting, qRT‐PCR and enzyme linked immunosorbent assay (ELISA) analyses (Figure 1P–S). Collectively, these findings indicate that FGF13 is decreased in the nigrostriatal tissues of both Parkinson's disease patients and parkinsonian mice.

### 2.2 FGF13 is Predominantly Expressed in Neurons and Decreased in Response to PD Neurotoxin

**Figure 2:**
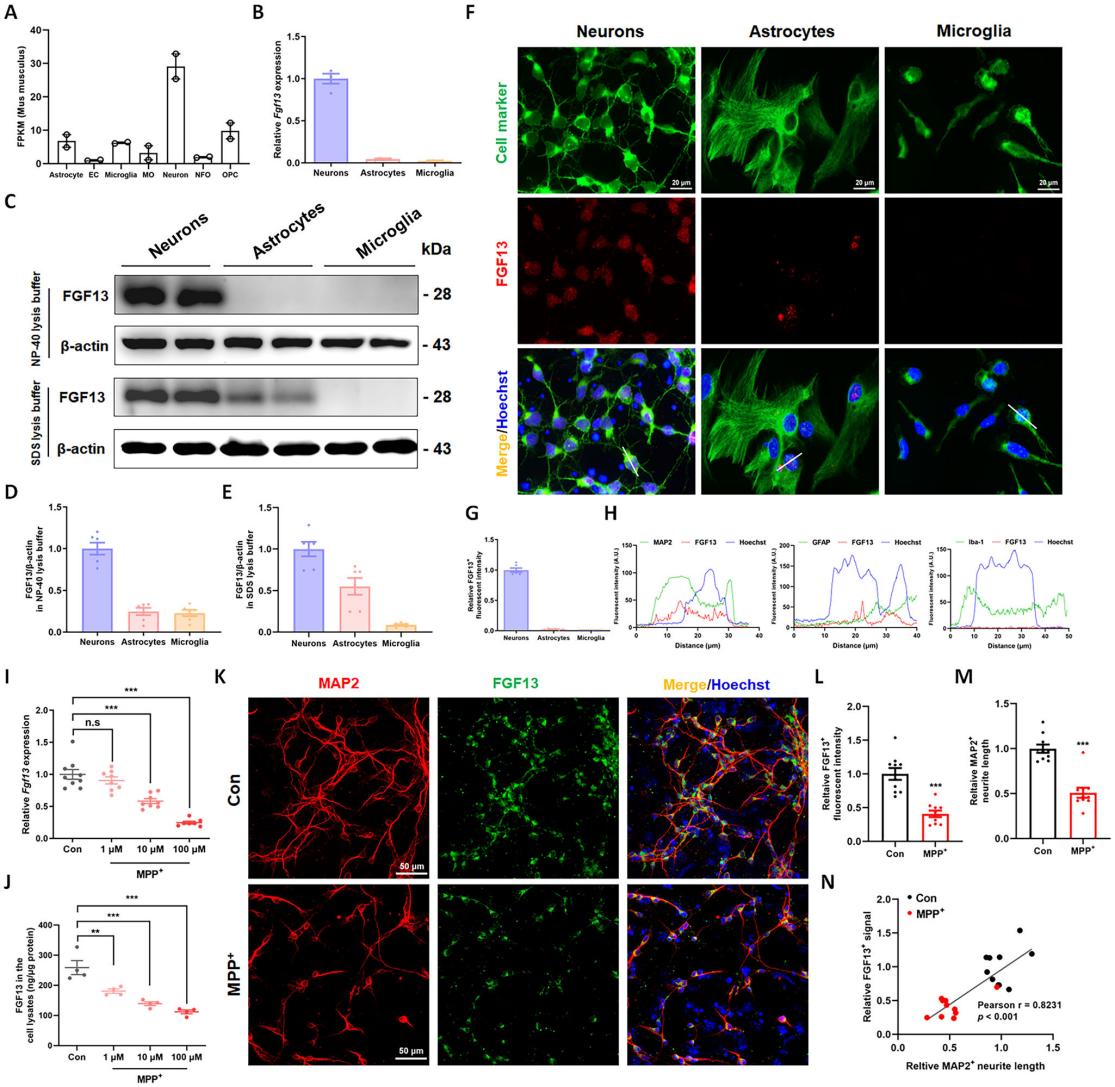
FGF13 is predominantly expressed in neurons and decreased in response to PD neurotoxin. A). *Fgf13* gene expression in different cell types of adult mouse cortex from a published RNA‐seq dataset (GSE52564: *n* = 2 for each group; EC means endothelial cell, MO means myelinating oligodendrocyte, NFO means newly formed oligodendrocyte and OPC means oligodendrocyte precursor). B). The *Fgf13* mRNA level in primary neurons, astrocytes, and microglia (*n* = 4 replicate experiments per group). C). FGF13 protein levels in lysates of primary cells lysed by NP‐40 buffer and SDS buffer. D). Quantitative analysis of FGF13 protein levels in cell lysates prepared using NP‐40 buffer (*n* = 6 replicate experiments per group). E). Quantitative analysis of FGF13 protein levels in cell lysates prepared using SDS buffer (n = 6 replicate experiments per group). F). Representative fluorescent images of FGF13 (red) and cell markers (green) in primary neurons marked by MAP2, astrocytes marked by GFAP, and microglia marked by Iba‐1. G). The relative fluorescent intensity of FGF13 in primary neural cells (6 images per group from 3 independent experiments). H). Co‐localization of FGF13 with cell body and nucleus in primary neurons, astrocytes, and microglia. I). *Fgf13* mRNA level in primary neurons upon MPP^+^ stimulation (*n* = 7–9 replicate experiments per group). J). FGF13 protein levels in lysates of primary neurons with MPP^+^ stimulation (*n* = 4 replicates per group). K). Representative fluorescent images of MAP2 (red) and FGF13 (green) in primary neurons stimulated with 10 µm MPP^+^ for 24 h. L). Quantitative analysis of FGF13‐positive signals in primary neurons (10 images per group from 3 independent experiments). M). Quantitative analysis of MAP2‐positive neurite length in primary neurons (10 images per group from 3 independent experiments). N). The correlation between the relative FGF13‐positive signals and the relative length of MAP2‐positive neurites in primary neurons stimulated with MPP^+^. All data are presented as the mean ± s.e.m. ^***^ *p* < 0.001; n.s means no significance. In I and J, a one‐way ANOVA with Dunnett's multiple comparisons test was used. In L and M, an unpaired two‐tailed Student's t‐test was used. In N, a Pearson correlation test was used.

We profiled *Fgf13* mRNA expression across multiple tissues in mice and reported that *Fgf13* was most abundantly expressed in the brain (Figure S2A, Supporting Information). By analyzing the published RNA‐seq data (GSE52564, GSE73721), *Fgf13* was found to be enriched in neurons and show relatively lower expression in non‐neuronal cells (**Figure** **2**A; Figure S2B, Supporting Information). To verify the cell‐specific expression of FGF13, we carried out primary cell culture experiments. It was demonstrated that *Fgf13* mRNA in neurons was far higher than that in astrocytes and microglia (Figure 2B). For the protein levels, FGF13 was highly expressed in neurons and was barely detectable in astrocytes and microglia by NP‐40 lysis buffer (Figure 2C,D). Interestingly, FGF13 was detectable in astrocytes, though its abundance was lower than that in neurons, by SDS lysis buffer that ruptures nuclear membranes (Figure 2C,E). Double‐labeling immunofluorescence further confirmed that FGF13 was highly expressed and widely distributed in neurons. In contrast, FGF13 was predominantly restricted to the nucleus in astrocytes (Figure 2F–H). Overall, FGF13 is predominantly expressed in primary neurons. Thus, we will focus our research on neuronal FGF13 and explore its implications in the pathogenesis of PD.

To examine the changes of FGF13 under PD stress in vitro, primary neurons were treated with MPP^+^ for 24 h. It found that *Fgf13* mRNA and FGF13 protein levels were decreased in a concentration‐dependent way (Figure 2I,J). The FGF13 levels in neuronal culture medium remained relatively low and did not change with increasing concentrations of MPP^+^ (Figure S2C, Supporting Information), indicating that MPP^+^ stimulation does not affect the secretion of FGF13. When we examined the FGF13 changes in damaged neurons, we observed reduced FGF13 fluorescence signals and shortened MAP2‐labeled processes in neurons with MPP^+^ stimulation (Figure 2K–M). Meanwhile, the process length and FGF13‐positive fluorescent intensity showed a positive correlation (Figure 2N). We also observed decreased FGF13‐positive fluorescent signal and sparse neuronal processes in α‐syn‐stimulated primary neurons (Figure S2D–F, Supporting Information), in which FGF13‐positive intensity was negatively correlated with the extent of neuronal damage (Figure S2G, Supporting Information). Together, these data suggest that FGF13 is highly expressed in neurons, and its expression decreases in primary neurons treated with PD neurotoxins.

### 2.3 Genetic Manipulation of Neuronal *Fgf13* Influences MPTP/p‐Induced Motor Dysfunctions and Dopaminergic Neuron Death

**Figure 3:**
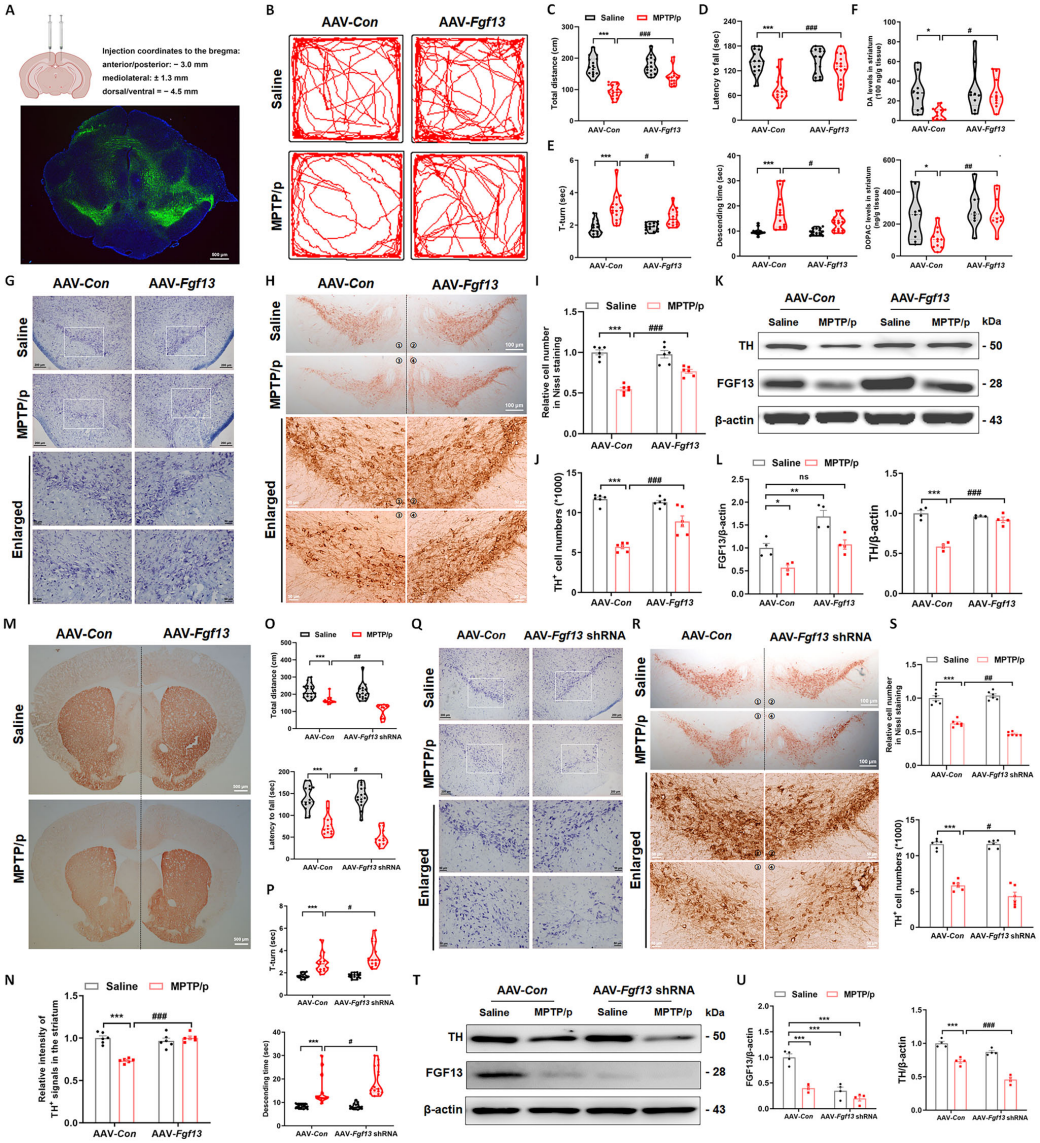
Genetic manipulation of neuronal *Fgf13* influences MPTP/p‐induced motor dysfunctions and dopaminergic neuron death. A). The strategy for injecting AAV into the SN of mice, along with the EGFP expression pattern in the midbrain after AAV injection. B). Representative movement tracks of mice in the OFT. C). The mean moving distance of mice in the OFT (*n* = 15 mice per group). D). The time that mice remained on the rotating rod in the rotarod test (*n* = 15 mice per group). E). The time taken by mice to turn around the top of the pole (T‐turn, left panel) and the time to descend the pole (descending time, right panel) in the pole test (*n* = 15 mice per group). F). Levels of dopamine and DOPAC in the striatum of mice (*n* = 9–11 mice; DOPAC means homoprotocatechuic acid). G). Representative images of Nissl staining in the SNc of mice. H). Representative immunohistological images of TH in the SNc of mice. I). Relative neuron numbers in the SNc of mice (*n* = 6 mice per group). J). Stereological counts of TH^+^ cells in the SNc (*n* = 6 mice per group). K). Representative immunoblots of TH and FGF13 in the midbrain. L). Quantitative analysis of FGF13 and TH proteins in immunoblotting (*n* = 4 mice per group). M). Representative immunohistological images of TH in the striatum of mice. N). Relative optical intensity of TH in the striatum of mice (*n* = 6 mice per group). O). The moving distance of mice in the OFT and the duration that mice stayed on the rotating rod in the rotarod test (*n* = 12–18 mice per group for the OFT and *n* = 15 for the rotarod test). P). T‐turn and descending time in the pole test (*n* = 15 mice per group). Q). Representative images of Nissl staining in the SNc. R). Representative immunohistological images of TH in the SNc of mice. S). The relative neuron numbers and stereological counts of TH‐positive cells in the SNc (*n* = 6 mice per group). T). Representative immunoblots of TH and FGF13 in the midbrain. U). Quantitative analysis of FGF13 and TH (*n* = 4 mice per group). All data are presented as the mean ± s.e.m. *p < 0.05, ^**^ *p* < 0.01, and ^***^ *p* < 0.001 versus. AAV‐*Con* Saline group; ^#^ *p* < 0.05, ^##^ *p* < 0.01, and ^###^ *p* < 0.001 versus. AAV‐*Con* MPTP/p group; n.s means no significance. Statistical comparison was performed using two‐way ANOVA.

To explore the effects of FGF13 on the pathological progression of PD, we injected a neuron‐specific *Fgf13*‐overexpressing adeno‐associated virus (AAV, with an hSyn promoter) into the midbrain to specifically increase *Fgf13* in mesencephalic neurons. The specificity of the virus was shown in Figure S3A (Supporting Information). Three weeks post‐injection, a large number of EGFP‐positive cells were observable in the midbrain (**Figure** **3**A). Subsequently, we established an MPTP/p‐induced PD mouse model (flowchart shown in Figure S3B, Supporting Information). After five and a half weeks of continuous modeling, voluntary movement behaviors were evaluated by the open field test (OFT), rotarod test, and pole test. In Figure 3B–E, chronic MPTP stimulation shortened the moving distance in the OFT and the latency time for falling from the rolling rod in the rotarod test, but prolonged the time for climbing over the rod tip (T‐turn) and descending the pole (descending time) in the pole test. These abnormal behavioral manifestations were significantly recovered in AAV‐*Fgf13* micro‐injected MPTP/p mice. We further measured striatal dopamine and its metabolite, dihydroxyphenyl acetic acid (DOPAC) levels by high‐performance liquid chromatography (HPLC), in which *Fgf13*‐overexpression significantly recovered the reduced levels of dopamine and its metabolite after MPTP/p stimulation (Figure 3F). Nissl staining was conducted in midbrain slices to mark the neurons, in which we observed that MPTP/p induced severe neuron loss in mice from vector‐control group and only a minor degree of neuron loss in *Fgf13*‐overexpressing mice (Figure 3G,I). In immunohistochemical staining of TH that marks dopaminergic neurons, over a half of the dopaminergic neurons in the midbrain (Figure 3H,J), and one‐third of TH‐positive fibers in the striatum (Figure 3M,N) were lost in MPTP/p‐treated AAV‐*Con* mice. However, these losses were significantly alleviated in MPTP/p‐treated *Fgf13*‐overexpressing mice. Consistently, *Fgf13* overexpression reversed the decreased TH protein in the midbrain of mice after chronic MPTP administration (Figure 3K,L). These data confirm that *Fgf13* up‐regulation in the SN mitigates MPTP/p‐induced behavioral phenotype and loss of nigrostriatal dopaminergic neurons in parkinsonian mice.

Neuropathological phenotypes in MPTP/p‐induced PD mice with mesencephalic *Fgf13* knockdown via AAV‐*Fgf13* short‐hairpin RNA (shRNA) micro‐injection were also evaluated. Compared with MPTP/p‐treated vector‐control mice, MPTP/p‐treated *Fgf13*‐knockdown mice showed a decreased moving distance in the OFT, a shorter latency to fall in the rotarod test (Figure 3O), and longer T‐turn and descending times in the pole test (Figure 3P). Meanwhile, *Fgf13* knockdown aggravated the loss of mesencephalic neurons and TH‐positive dopaminergic neurons induced by chronic MPTP stimulation (Figure. 3Q–U). We further supplemented FGF13 protein by local micro‐injection into the midbrain and then established acute MPTP‐induced PD mice model, and the modeling process was illustrated in Figure S3C (Supporting Information). Interestingly, we observed that FGF13 supplementation showed no effect on the loss of nigrostriatal neurons, demonstrated by the absence of significant differences in the numbers of mesencephalic neurons (Figure S3D,F, Supporting Information), the numbers of TH‐positive neurons (Figure S3E,G, Supporting Information), TH protein levels (Figure S3H,I, Supporting Information), and the optical density of striatal TH‐positive fibers (Figure S3J,K, Supporting Information) between the MPTP group and the FGF13 plus MPTP group. These results strongly emphasize that, despite the potential for FGF13 to be secreted, it does not contribute to the pathogenesis of PD via membranous receptor‐based extracellular signaling mechanisms. Therefore, genetic regulation of neuronal *Fgf13* influences MPTP/p‐induced motor dysfunction and dopaminergic neuron death.

### 2.4 Neuronal FGF13 Regulates Neuroinflammation and Glial Reactivity in the Nigrostriatal Pathway

**Figure 4:**
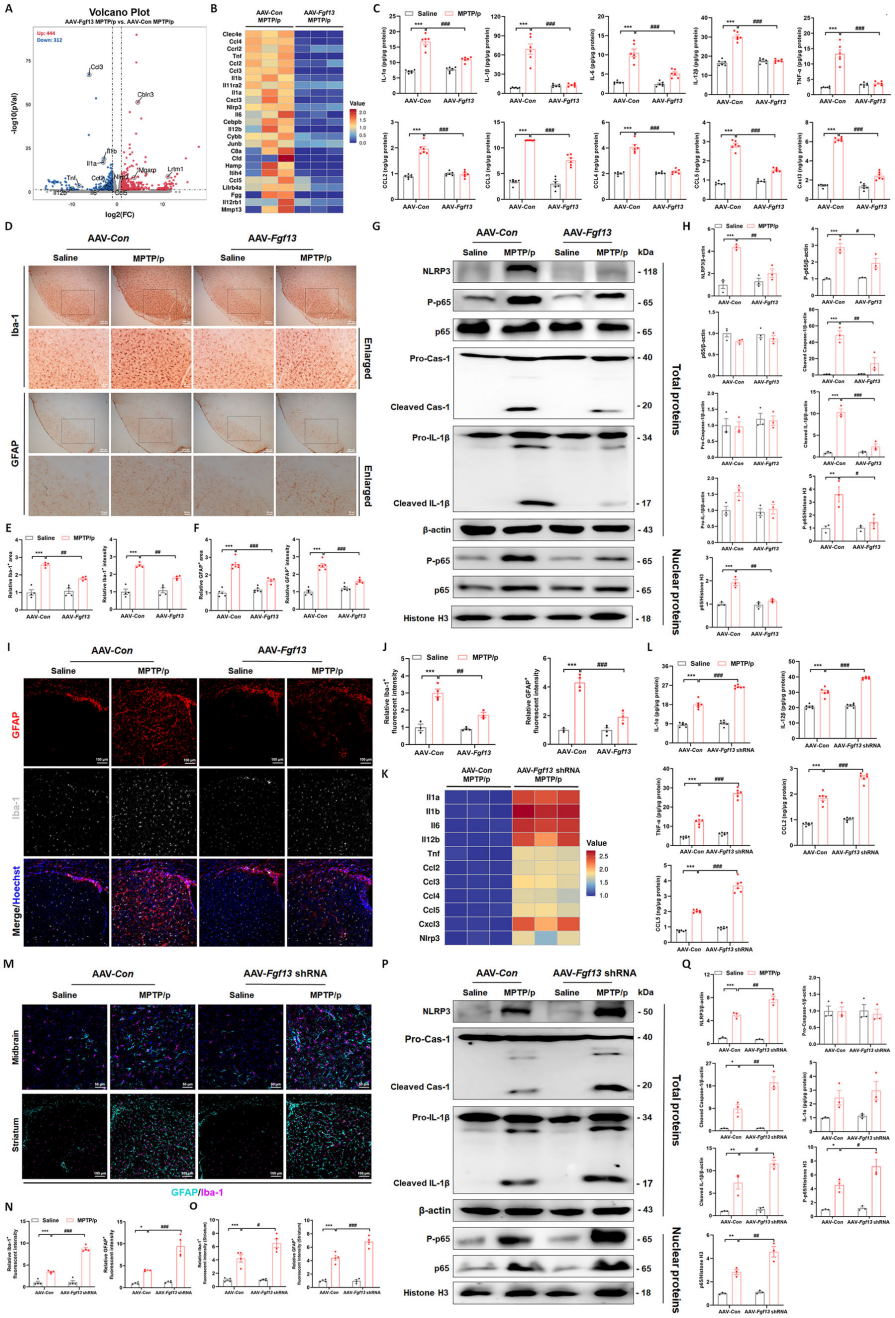
Neuronal FGF13 regulates neuroinflammation and glial reactivity in the nigrostriatal pathway. A). Volcano plot of the DEGs (|log2 fold change| ≥ 1 and adjusted *P* < 0.05) in AAV‐*Fgf13* MPTP/p versus. AAV‐*Con* MPTP/p was identified through RNA‐sequencing analysis of nigrostriatal tissues. B). Heat map presenting the expression of inflammatory genes and chemokines identified by RNA‐seq. C). Levels of inflammatory cytokines and chemokines in the lysates of nigrostriatal tissues by ELISA assay (*n* = 6 mice per group). D). Representative immunohistological images of Iba‐1 and GFAP in the SNc of mice. E). Quantitative analysis of Iba‐1‐positive area and intensity in the SNc of mice (*n* = 4 mice per group). F). Quantitative analysis of GFAP‐positive area and intensity in the SNc of mice (*n* = 5–6 mice per group). G). Representative immunoblots of NLRP3, phosphorylated p65 (P‐p65), p65, Caspase‐1 (Cas‐1), and IL‐1β in total lysates; as well as P‐p65 and p65 in the nuclear lysates of the midbrain tissues. H. Quantitative analysis of NLRP3, P‐p65, p65, Cas‐1, and IL‐1β in total lysates; and P‐p65 and p65 in nuclear lysates in the immunoblotting experiment (*n* = 3 replicates per group). I. Representative fluorescent images of GFAP (red) and Iba‐1 (grey) in the striatum of mice. J). Quantitative analysis of Iba‐1‐ and GFAP‐positive fluorescent signals in the striatum (*n* = 3–4 mice per group). K). Heat map presenting the expression of inflammatory genes and chemokines in the midbrain, as determined by qRT‐PCR. L). Levels of inflammatory cytokines and chemokines in the lysates of mesencephalic tissues by ELISA assay (*n* = 6 mice per group). M). Representative fluorescent images of GFAP (turquoise) and Iba‐1 (purple) in the midbrain (upper panel) and in the striatum (lower panel). N). Quantitative analysis of Iba‐1‐ and GFAP‐positive fluorescent signals in the midbrain (*n* = 4 mice per group). O). Quantitative analysis of Iba‐1‐ and GFAP‐positive fluorescent signals in the striatum (*n* = 4 mice per group). P). Representative immunoblots of NLRP3, Cas‐1, and IL‐1β in total lysates; as well as P‐p65 and p65 in the nuclear lysates of the midbrain tissues. Q). Quantitative analysis of NLRP3, P‐p65, p65, Cas‐1, and IL‐1β in total lysates; and P‐p65 and p65 in nuclear lysates in the immunoblotting experiments (*n* = 3 replicates per group). All data are presented as the mean ± s.e.m. ^*^ *p* < 0.05, ^**^ *p* < 0.01, and ^***^ *p* < 0.001 versus. AAV‐*Con* Saline group; ^#^ *p* < 0.05 and ^##^ *p* < 0.01, and ^###^ *p* < 0.001 versus. AAV‐*Con* MPTP/p group. Statistical comparison was performed using two‐way ANOVA.

To further investigate the role of FGF13 in neuronal fate under PD‐related stress, primary neurons from the midbrain were transfected with lentivirus (LV) packaged with *Fgf13* plasmids and then treated with MPP^+^. We unexpectedly found that *Fgf13* overexpression via lentivirus (Lenti‐*Fgf13*) failed to improve the decreased neuronal cell viability caused by MPP^+^ ranging from 1 to 100 µm within 24 h (Figure S4A–C, Supporting Information). Specifically, *Fgf13* overexpression showed no effect on the decreased neurite length and reduced live cell number induced by 10 µM MPP^+^ (FigureS4D–G, Supporting Information). Subsequently, neurons were pre‐treated with FGF13 protein (10 ng·mL^−1^), and then treated with MPP^+^. It was also found that FGF13 supplementation showed no effects on neuronal damage (Figure S4H–J, Supporting Information). These in vitro results, though inconsistent with those from PD mouse model, may provide valuable insights for subsequent mechanism exploration.

Next, we attempted to gain deeper insight into how FGF13 protected dopaminergic neuron loss in vivo but showed no effects in vitro. We therefore collected the midbrain and striatum tissues from AAV‐*Con* MPTP/p and AAV‐*Fgf13* MPTP/p mice for RNA sequencing. A total of 756 DEGs were identified using the criteria of |log_2_ fold change| ≥ 1 and adjusted P < 0.05. Among them, 444 genes were up‐regulated and 312 genes were down‐regulated, when comparing the AAV‐*Fgf13* MPTP/p group with the AAV‐*Con* MPTP/p group, as illustrated in **Figure** **4**A. From gene set enrichment analysis, DEGs were enriched in multiple inflammatory pathways, such as NOD‐like receptor signaling pathway, TNF signaling pathway, and IL‐17 signaling pathway by KEGG pathway enrichment analysis, or immune response and immune system process by GO functional enrichment (Figure. S4K,L, Supporting Information). We analyzed the levels of inflammatory genes and chemokine genes based on the RNA‐seq data, and we found that these genes displayed a general downward trend in the AAV‐*Fgf13* plus MPTP/p group compared to the AAV‐*Con* plus MPTP/p group (Figure 4B). Furthermore, *Fgf13* overexpression effectively inhibited the elevated levels of inflammatory cytokines and chemokines in total nigrostriatal lysates of MPTP/p mice (Figure 4C). Inflammatory events in the CNS are instigated by the misfiring of immune cells, among which microglia and astrocytes act as pivotal cell types.^[^ ^4^, ^5^ ^]^ We therefore performed immunohistochemical analysis of microglia (Iba‐1) and astroglia (GFAP) in midbrain slices. In Figure 4D–F, chronic MPTP stimulation induced significant increase in the area and intensity of Iba‐1‐ and GFAP‐positive signals, which were markedly reversed by *Fgf13* overexpression. Moreover, AAV‐*Fgf13* micro‐injection inhibited the activation of NF‐κB signaling pathway (including p65 phosphorylation, nuclear translocation of p65, and phosphorylated p65), as well as the increase of NLRP3 inflammasome mediators (including NLRP3, cleaved IL‐1β and cleaved Caspase‐1) in the midbrain of MPTP/p‐treated mice (Figure 4G,H). Similarly, the inhibitory effects of *Fgf13* overexpression on glial activation were also evidently observed in the striatal region (Figure 4I,J). Furthermore, the neuroinflammatory responses and glial reactivity in MPTP/p mice following *Fgf13* down‐regulation were evaluated. It was found that *Fgf13* loss‐of‐function via AAV‐*Fgf13* shRNA significantly increased the levels of inflammatory cytokines and chemokines in the midbrain under chronic MPTP stimulation (Figure 4K,L). Additionally, *Fgf13* interference aggravated the activation of microglia and astrocytes in both the midbrain and striatum (Figure 4M–O). This was accompanied by intensified activation of the NF‐κB signaling cascade and the downstream NLRP3 inflammasome molecules (Figure 4P,Q). Taken together, neuronal FGF13 rescues MPTP/p‐induced neuroinflammation and glial reactivity in the nigrostriatal pathway. Consequently, it is reasonable to postulate that glial cells may be essential for the role of neuronal FGF13 in the progression of PD.

### 2.5 FGF13 Modulates Neuronal Damage and Glial Inflammatory Events in a Neuron‐Glia Co‐Culture System

**Figure 5:**
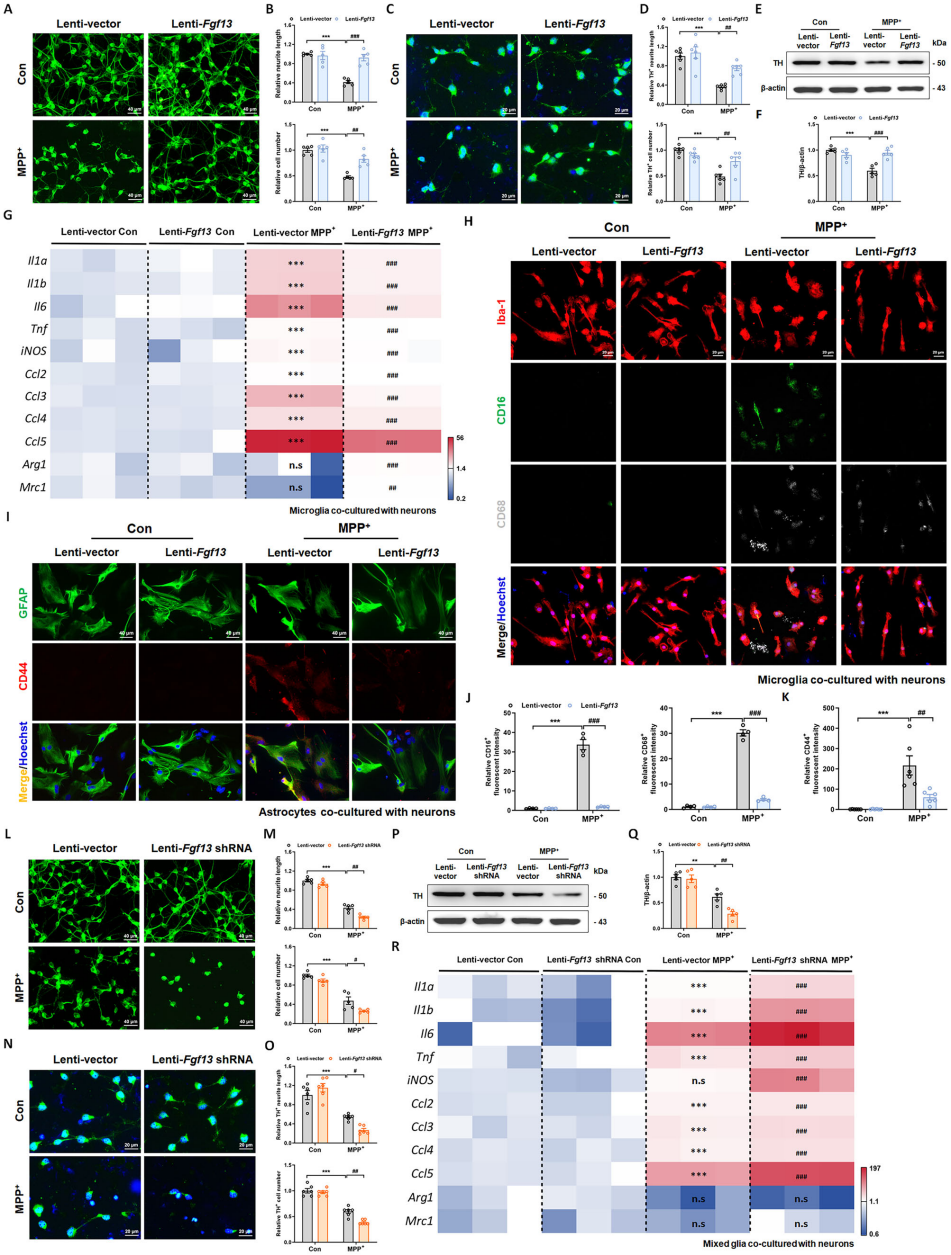
FGF13 modulates neuronal damage and glial inflammatory events in a neuron‐glia co‐culture system. A). Representative fluorescent images of MAP2 (green) in primary neurons. B). The relative neurite length and cell number in MAP2‐positive neurons (5 images). C). Representative fluorescent images of TH (green) in primary neurons. D). The relative neurite length and cell number in TH‐positive dopaminergic neurons (6 images from 3 independent experiments). E). Representative immunoblots of TH in primary neurons. F). Quantitative analysis of TH in the immunoblotting experiments (*n* = 5 replicates per group). G). Heat map presenting the levels of inflammatory genes and chemokines in primary microglia by qRT‐PCR assay (*n* = 3 replicates per group). H). Representative fluorescent images of Iba‐1 (red), CD16 (green, marker of M1‐polarized microglia), and CD68 (grey, marker of activated microglia) in primary microglia. I). Representative fluorescent images of GFAP (green) and CD44 (red, marker of activated astrocytes) in primary astrocytes. J). The relative fluorescent intensities of CD16 (left panel) and CD68 (right panel) in primary microglia (4 images). K). The relative fluorescent intensity of CD44 in primary astrocytes (6 images from 3 independent experiments). L). Representative fluorescent images of MAP2 (green) in primary neurons. M). The relative neurite length and cell number in MAP2‐positive neurons (5 images). N). The representative fluorescent images of TH (green) in primary neurons. O). Relative neurite length and cell number in TH‐positive dopaminergic neurons (6 images from 3 independent experiments). P). Representative immunoblots of TH in primary neurons. Q). Quantitative analysis of TH in the immunoblotting experiments (*n* = 5 replicates per group). R). Heat map presenting the levels of inflammatory genes and chemokines in the mixed glial cultures by qRT‐PCR assay (*n* = 3 replicates per group). All data are presented as the mean ± s.e.m. ^**^ *p* < 0.01 and ^***^ *p* < 0.001 versus. Lenti‐vector Con group; ^#^ *p* < 0.01, ^##^ *p* < 0.01, and ^###^ *p* < 0.001 versus. Lenti‐vector MPP^+^ group; n.s means no significance. Statistical comparison was performed using two‐way ANOVA.

To verify our speculation, a neuron‐glia co‐culture system was performed in transwell chambers as the schematic diagrams presented (Figure S5A–C, Supporting Information). We observed that neurons overexpressing *Fgf13*, when co‐cultured with glial cells, exhibited resistance to MPP^+^‐induced decrease in MAP2‐positive neurite length and live cell numbers (**Figure** **5**A,B). Similar results were obtained in neurons stimulated with α‐syn in the neuron‐glia co‐culture system (Figure S5D–F, Supporting Information). Furthermore, dopaminergic neurons overexpressing *Fgf13* exhibited enhanced resistance to MPP^+^ toxicity. This was evidenced by increased TH‐positive neurite length, elevated TH‐positive cell numbers (Figure 5C,D), and higher TH protein levels (Figure 5E,F) in dopaminergic neurons co‐cultured with glia. We simultaneously evaluated the inflammatory responses of glia in the co‐culture system. Microglia co‐cultured with neurons of Lenti‐vector plus MPP^+^ group exhibited increased expression of pro‐inflammatory and chemokine genes, whereas those co‐cultured with neurons of Lenti‐*Fgf13* plus MPP^+^ group showed attenuated effects (Figure 5G). Immunofluorescent co‐labeling of Iba‐1, CD68 (marker of activated microglia), and CD16 (marker of M1‐polarized microglia) further demonstrated the inhibitory effects of *Fgf13* overexpression on microglia activation (Figure 5H,J). Astrocytes co‐cultured with *Fgf13*‐overexpressing neurons exhibited significantly attenuated CD44 immunoreactivity in response to MPP^+^ challenge (Figure 5I,K), indicating suppression of astrocytic reactivity. *Fgf13* inhibition by Lenti‐*Fgf13* shRNA aggravated neuronal injuries induced by MPP^+^ in the neuron‐glia co‐culture system (Figure 5L,M). Consistently, *Fgf13* knockdown aggravated the loss of dopaminergic neurons induced by MPP^+^ (Figure 5N–Q). Moreover, *Fgf13* down‐regulation further increased gene levels of pro‐inflammatory and chemokine genes in the mixed glial cultures (Figure 5R). We therefore conclude that FGF13 modulates neuronal damage and glial inflammatory events in the neuron‐glia co‐culture system.

### 2.6 FGF13 Restrains Mitochondrial Release from Neurons

**Figure 6:**
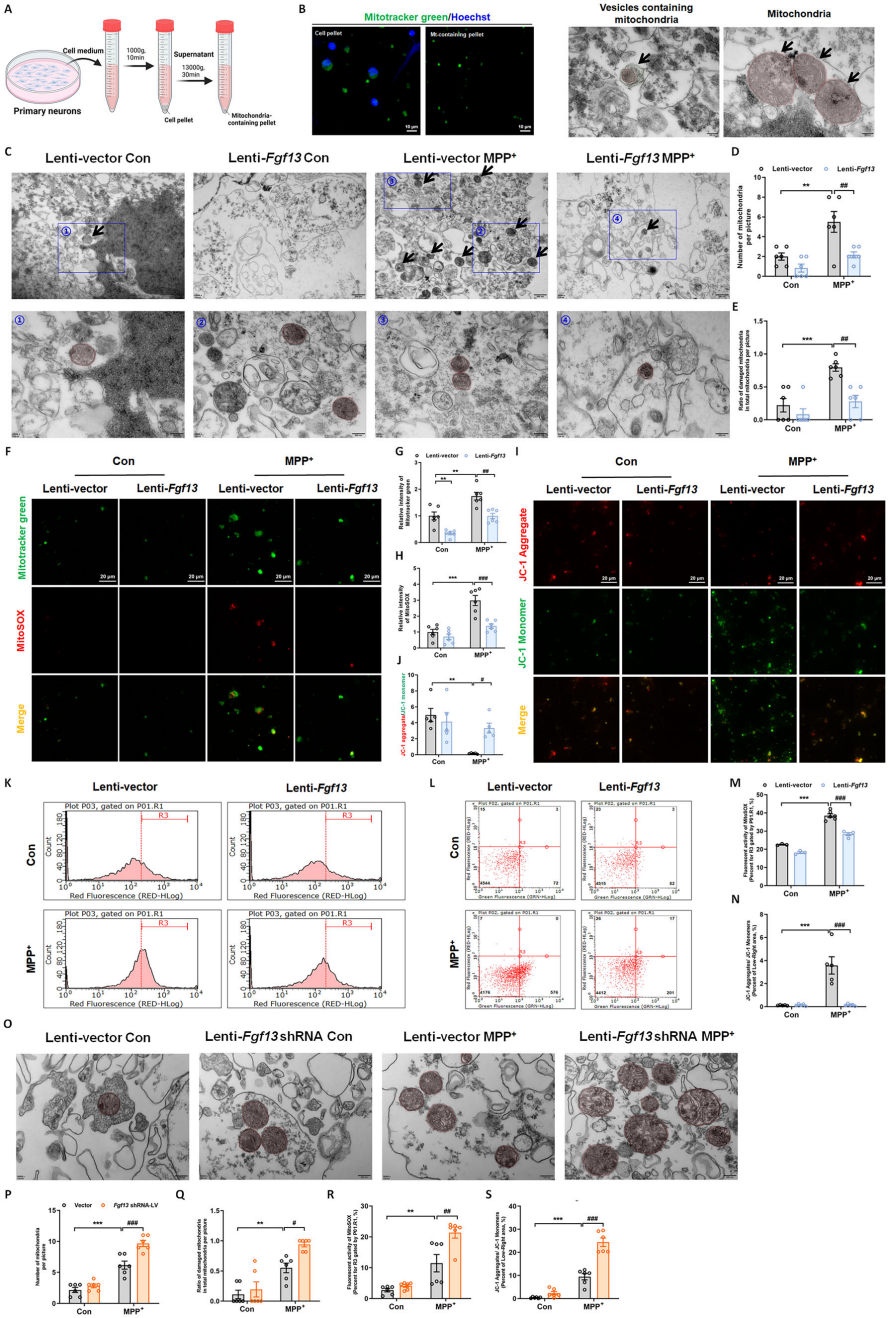
FGF13 restrains mitochondrial release from neurons. A). Experimental schematic for collecting extracellular mitochondria from neuron‐conditioned medium via differential ultracentrifugation. B). Left panel: representative fluorescent image of intracellular and extracellular mitochondria (Mitotracker green labels mitochondria, Hoechst labels the nuclei). Right panel: representative TEM images showing free mitochondria (pink circles) and vesicles (green circle) that contain mitochondria (pink circles). C). Representative TEM images of extracellular mitochondria. D). Quantitative analysis of extracellular mitochondria per picture (6 pictures from 3 replicates per group). E). The percentage of damaged mitochondria among all mitochondria in each picture (6 pictures from 3 replicates per group). F). Representative fluorescent images of extracellular mitochondria stained with Mitotracker green and MitoSOX Red. G). Quantitative analysis of Mitotracker green signals (6 images from 3 independent experiments). H. Quantitative analysis of MitoSOX Red signals (6 images from 3 independent experiments). I). Representative fluorescent images of extracellular mitochondria stained with JC‐1. J). The ratio of JC‐1 aggregate to JC‐1 monomer in extracellular mitochondria (5 images from 5 independent experiments). K). MitoSOX Red signals analyzed by flow cytometry. L. Ratio of JC‐1 aggregate to JC‐1 monomer in extracellular mitochondria analyzed by flow cytometry. M). Quantitative analysis of MitoSOX Red signals in flow cytometry (*n* = 3–6 replicates per group). N). Quantitative analysis of JC‐1 aggregate/JC‐1 monomer in flow cytometry (*n* = 5 replicates per group). O). Representative TEM images of extracellular mitochondria. P). Quantitative analysis of extracellular mitochondria per picture (6 pictures from 3 replicates per group). Q). The percentage of damaged mitochondria among all mitochondria in each picture (6 pictures from 3 replicates per group). R). Quantitative analysis of MitoSOX Red signals in flow cytometry (*n* = 6 replicates per group). S). Quantitative analysis of JC‐1 aggregate/JC‐1 monomer in flow cytometry (*n* = 6 replicates per group). All data are presented as the mean ± s.e.m. ^*^ *p* < 0.05, ^**^ *p* < 0.01, and ^***^ *p* < 0.001 versus. Lenti‐vector Con group; ^#^ *p* < 0.05, ^##^ *p* < 0.01, and ^###^ *p* < 0.001 versus. Lenti‐vector MPP^+^ group; n.s means no significance. Statistical comparison was performed using two‐way ANOVA.

Given that neuronal FGF13 only determines neuron susceptibility in the neuron‐glia co‐culture system, we speculate that FGF13 regulates the interplay between neurons and glia via unknown mechanisms. Recently, extracellular release of mitochondria has been reported to mediate mitochondrial quality control, sustain micro‐environment homeostasis, and facilitate cell communication.^[^ ^20^, ^21^ ^]^ Particularly, these mitochondria or mitochondria‐containing vesicles are released to surroundings of donor cells and evoke immune responses in target cells.^[^ ^21^ ^]^ In our study, we used a differential centrifugation method to isolate mitochondria from cell medium and confirmed their presence in health primary neuron cultures (**Figure** **6**A,B). Transmission electron microscopy (TEM) revealed that MPP^+^ increased the number of mitochondria and the proportion of damaged mitochondria in the culture medium, while *Fgf13* overexpression reversed these effects (Figure 6C–E). We also used a mitochondria‐selective probe (Mitotracker green), a fluorescent probe staining mitochondrial superoxide (MitoSOX) and a mitochondrial membrane potential probe (JC‐1) to assess the number and quality of the released mitochondria. It was shown that MPP^+^ treatment increased the efflux of both normal mitochondria and stressed mitochondria, which was partially restored in the Lenti‐*Fgf13* plus MPP^+^ group (Figure 6F–H,K,M). It should be noted that Lenti‐*Fgf13* alone reduced the level of extracellular healthy mitochondria (Figure 6F,G). In the representative images and flow cytometric analysis of JC‐1 for the mitochondrial membrane potential assay (Figure 6I,J,L,N), overexpression of *Fgf13* reversed the transformation of JC‐1 monomers (green fluorescent signals) to aggregates (red fluorescent signals). This reversal effect indicated that overexpression of *Fgf13* counteracted the mitochondrial depolarization induced by MPP^+^. For cytoplasmic mitochondria in neurons, we observed no significant change of mitochondrial mass and functions in MPP^+^‐treated primary neurons transfected with either Lenti‐vector or Lenti‐*Fgf13* (Figure S6A–F, Supporting Information). Our findings further demonstrated that the overexpression of *Fgf13* not only suppressed the extracellular release of mitochondria, but also mitigated the super‐oxidation and depolarization of extracellular mitochondria induced by α‐syn (Figure S6G–J, Supporting Information). Moreover, *Fgf13* knockdown via Lenti‐*Fgf13* shRNA transfection induced mild mitochondrial efflux under normal conditions, although this effect was not statistically significant. Nonetheless, upon MPP^+^ stimulation, *Fgf13* knockdown significantly enhanced the release of mitochondria (Figure 6O–Q). Additionally, the percentage of depolarized mitochondria and mitochondria with superoxide was significantly higher in the Lenti‐*Fgf13* shRNA plus MPP^+^ group than that of the Lenti‐vector plus MPP^+^ group (Figure 6R,S). Accordingly, FGF13 inhibits the release of both healthy and stressful mitochondria from neurons.

### 2.7 Neuronal FGF13 Modulates Inflammatory Responses in Glia via Regulating Mitochondrial Transfer

**Figure 7:**
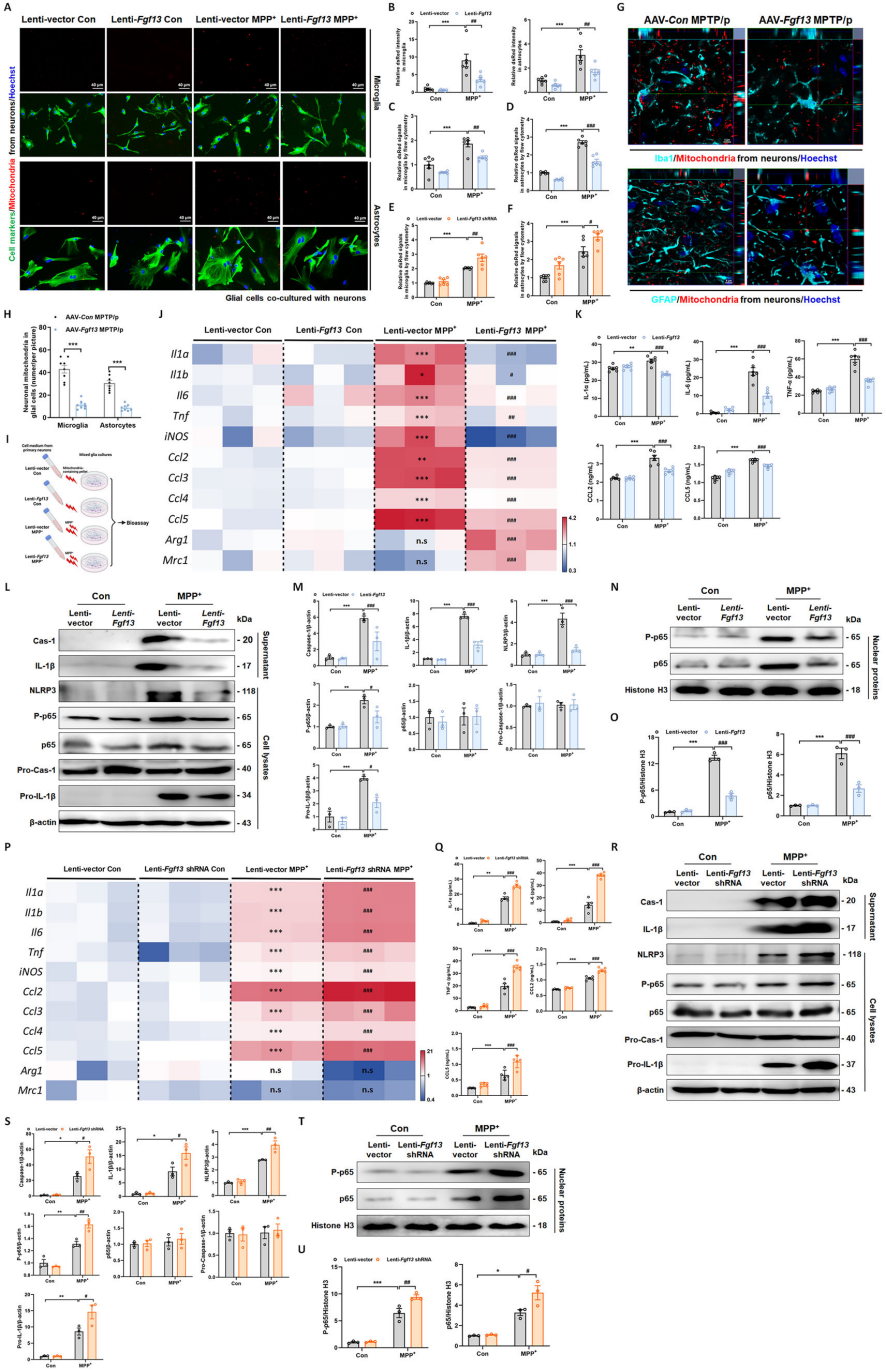
Neuronal FGF13 modulates inflammatory responses in glia via regulating mitochondrial transfer. A). Representative fluorescent images of neuronal mitochondria (marked by Mito‐dsRed) and Ibal (green, upper panel), as well as GFAP (green, lower panel) in cell cultures. B). Quantitative analysis of neuronal mitochonria in microglia (left panel) and astrocytes (right panel) (6 images from 3 independent experiments). C). Quantitative analysis of Mito‐dsRed signals in microglia stimulated with mitochondria from *Fgf13*‐overexpressing neurons by flow cytometric analysis (*n* = 6 replicates per group). D). Quantitative analysis of Mito‐dsRed signals in astrocytes stimulated with mitochondria from *Fgf13*‐overexpressing neurons (*n* = 6 replicates per group). E). Quantitative analysis of Mito‐dsRed signals in microglia stimulated with mitochondria from *Fgf13*‐knockdown neurons (*n* = 6 replicates per group). F). Quantitative analysis of Mito‐dsRed signals in astrocytes stimulated with mitochondria from *Fgf13*‐knockdown neurons (*n* = 6 replicates per group). G). Representative fluorescent images of neuronal mitochondria (marked by Mito‐dsRed) and Ibal (turquoise, upper panel), as well as GFAP (turquoise, lower panel) in the midbrain slices. H). Quantitative analysis of neuronal mitochonria in microglia and astrocytes (right panel) (8 images from 4 independent experiments). I). Schematic diagram illustrating the treatment of mixed glial cultures with mitochondria collected from neuronal culture medium. J). Heat map presenting the levels of inflammatory genes and chemokines in mixed glial cultures (*n* = 3 replicates per group). K). Levels of inflammatory cytokines and chemokines in the surpernatant of mixed glial culture (*n* = 6 replicates per group). L. Representative immunoblots of NLRP3, P‐p65, p65, pro‐Cas‐1, and pro‐IL‐1β in total lysates; as well as Cas‐1 and IL‐1β in the surpernatant. M). Quantitative analysis of NLRP3, P‐p65, p65, Cas‐1 and IL‐1β (*n* = 3 replicates per group). N. Representative immunoblots of P‐p65 and p65 in the nuclear lysates of glial cultures. O). Quantitative analysis of P‐p65 and p65 in the nuclear lysates (*n* = 3 replicates per group). P). Heat map presenting the levels of inflammatory genes and chemokines in mixed glial cultures (*n* = 3 replicates per group). Q). Levels of inflammatory cytokines and chemokines in the surpernatant of mixed glial culture (n = 6 replicates per group). R). Representative immunoblots of NLRP3, P‐p65, p65, pro‐Cas‐1 and pro‐IL‐1β in total lysates; as well as Cas‐1 and IL‐1β in the surpernatant. S). Quantitative analysis of NLRP3, P‐p65, p65, Cas‐1 and IL‐1β (*n* = 3 replicates per group). T). Representative immunoblots of P‐p65 and p65 in the nuclear lysates of glial cultures. U). Quantitative analysis of P‐p65 and p65 (*n* = 3 replicates per group). All data are presented as the mean ± s.e.m. For H, ^***^ *p* < 0.001 versus. AAV‐*Con* MPTP/p group. For other panels, ^*^ *p* < 0.05, ^**^ *p* < 0.01, and ^***^ *p* < 0.001 versus. Lenti‐vector Con group; ^#^ *p* < 0.05, ^##^ *p* < 0.01, and ^###^ *p* < 0.001 versus. Lenti‐vector MPP^+^ group; n.s means no significance. Statistical comparison was performed using two‐way ANOVA.

To probe into whether the mitochondria released from neurons were transferred to glial cells, we transfected primary neurons with Mito‐dsRed lentivirus to label the mitochondria. Subsequently, the labeled neurons were co‐cultured separately with microglia and astrocytes (Figure S7A, Supporting Information). We observed the presence of neuronal mitochondrial particles in glial cell cultures (**Figure** **7**A). Specifically, microglia and astrocytes co‐cultured with neurons of the Lenti‐*Fgf13* plus MPP^+^ group exhibited decreased uptake of neuronal mitochondria compared to those co‐cultured with neurons of the Lenti‐vector plus MPP^+^ group (Figure 7A,B). Flow cytometric analysis consistently showed that the uptake of neuronal mitochondria by microglia and astrocytes significantly decreased in the Lenti‐*Fgf13* plus MPP^+^ group (Figure 7C,D). Meanwhile, glial cells co‐cultured with neurons of the Lenti‐*Fgf13* shRNA plus MPP^+^ group took up more neuronal mitochondria than those of the Lenti‐vector plus MPP^+^ group (Figure 7E,F). We further investigated the regulatory effects of FGF13 on neuronal mitochondrial efflux and mitochondrial transfer to glial cells in vivo. Neuronal mitochondria in the midbrain were visualized by micro‐injection of rAAV‐hSyn‐Mito‐dsRed, and the MPTP/p mouse model was subsequently established (Figure S7B,C, Supporting Information). Chronic MPTP administration triggered significant mitochondrial efflux from midbrain neurons, which was substantially attenuated by *Fgf13* overexpression (Figure S7D,E, Supporting Information). Furthermore, MPTP/p mice with *Fgf13* overexpression exhibited a decrease in neuron‐derived mitochondria in both microglia and astrocytes (Figure 7G,H), suggesting that *Fgf13* overexpression inhibits neuronal mitochondria transfer to glial cells under PD stress.

We then further investigated whether these neuron‐derived mitochondria regulated the inflammatory responses in adjacent glia. To this end, isolated mitochondria from neuronal medium were employed to stimulate the glial cultures. During this process, glial cells were also treated with PD neurotoxin in the groups receiving isolated mitochondria from neurons under PD‐like stress, to more accurately mimic the in vivo environment (Figure 7I). Neuronal mitochondria from the MPP^+^ group, in combination with MPP^+^ stimulation, provoked a significant increase of pro‐inflammatory cytokines and chemokines in mixed glial cells. However, neuronal *Fgf13* overexpression effectively inhibited these inflammatory effects in glial cells, as confirmed by qRT‐PCR and ELISA analyses (Figure 7J,K). Consistent with these findings, neuronal overexpression of *Fgf13* effectively suppressed both the NF‐κB signaling cascade and downstream NLRP3 inflammasome activation in glial cells (Figure 7L–O). *Fgf13* knockdown experiments were also assessed, revealing that neuronal *Fgf13* loss‐of‐function exacerbated the inflammatory responses in glial cells by qRT‐PCR, ELISA, and immunoblotting analyses (Figure 7P–U).

To eliminate the potential impact of other inflammatory factors that may exist in the neuronal culture medium, we performed subsequent flow cytometric sorting to purify neuronal mitochondria. The purified mitochondria were then utilized to stimulate glial cultures (Figure S7F,G, Supporting Information). The approximate quantity of sorted mitochondria is shown in Figure S7H (Supporting Information). We observed that mitochondria from MPP^+^‐treated neurons evidently activated the pro‐inflammatory and chemokine genes in glia, and neuronal *Fgf13* overexpression significantly suppressed the expression of most of the genes (Figure S7I, Supporting Information). Conversely, *Fgf13* knockdown in neurons markedly increased the expression of most of these genes (Figure S7J, Supporting Information). Taken together, neuronal *Fgf13* overexpression modulates inflammatory signals in glia by regulating mitochondrial transfer.

### 2.8 FGF13 Interacts with Mitochondrial Proteins to Prohibit Mitochondrial Leakage

**Figure 8:**
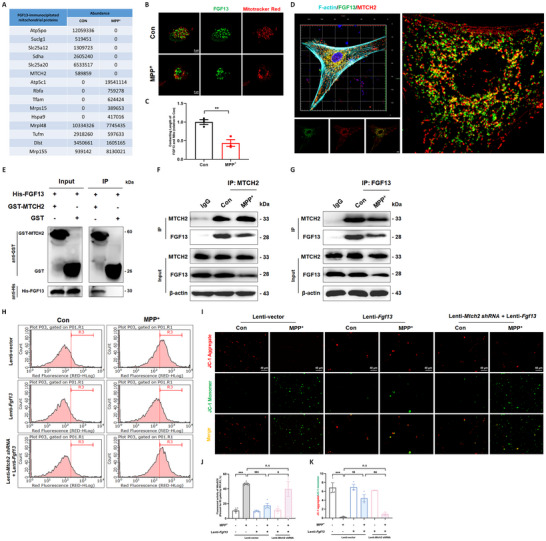
FGF13 interacts with mitochondrial proteins to prohibit mitochondrial leakage. A). Abundance of FGF13‐binding mitochondrial proteins in primary neurons assessed by mass spectrometry. B). Representative fluorescent images of FGF13 (green) and Mitotracker Red in primary neurons. C). The contacting length of FGF13 and mitochondria in the primary neurons. D). Co‐localization of FGF13 (green) and MTCH2 (red) in the HEK293T cell, with the cytoskeleton (F‐actin) visualized using phalloidin staining (turquoise). E). Detection of His‐FGF13 bound to GST‐MTCH2 or GST in a GST pull‐down assay. F–G). Co‐immunoprecipitation assay of FGF13 and MTCH2 in primary neurons. H). MitoSOX Red signals in the mitochondria‐containing pellets were analyzed by flow cytometry. I). Representative fluorescent images of extracellular mitochondria stained with JC‐1. J). Quantitative analysis of MitoSOX Red signals in the flow cytometry assay (*n* = 4–6 replicates per group). K). Ratio of JC‐1 aggregate to JC‐1 monomer in extracellular mitochondria (from 3 independent experiments). All data are presented as the mean ± s.e.m. For C, ^**^ *p* < 0.01 versus. Con group. For other panels, ^***^ *p* < 0.001 versus. Lenti‐vector Con group; $$*p* < 0.01 and $$$*p* < 0.001 versus. Lenti‐vector MPP^+^ group; &*p* < 0.05 and &&*p* < 0.01 versus. Lenti‐*Fgf13* MPP^+^ group; n.s means no significance. Statistical comparison was performed using one‐way ANOVA with Dunnett's multiple comparisons test.

Previous studies have identified multiple FGF13‐binding proteins, by which FGF13 regulates neural stem cell development, neuronal polarization and migration.^[^ ^22^, ^23^ ^]^ Here in our study, we screened for FGF13‐binding protein in control and MPP^+^‐treated neurons by mass spectrum analysis of the immune sedimentation component (schematic diagram shown in Figure S8A, Supporting Information). We identified several FGF13 partners that have been previously reported including microtubule associated proteins and mitogen‐activated protein kinase.^[^ ^14^, ^22^ ^]^ Moreover, multiple mitochondrial proteins were found to interact with FGF13, and most of these proteins decreased in MPP^+^ group (**Figure** **8**A). Further experiments verified the localization of FGF13 in the vicinity of mitochondria (Figure 8B). Specifically, in MPP^+^‐treated neurons, FGF13 signals around mitochondria were sparse (Figure 8B,C). Among these FGF13‐binding mitochondrial proteins, we had a special focus on MTCH2 (whose mass spectrum was shown in Figure S8B, Supporting Information) for its localization on the mitochondrial outer membrane and the function as a gatekeeper for the outer membrane.^[^ ^24^ ^]^ After co‐transfection of *Fgf13* and *Mtch2* plasmids, we observed FGF13 interacted with MTCH2 within HEK293T cells (Figure 8D). We further demonstrated the direct interaction between FGF13 and MTCH2 using glutathione S‐transferase (GST) pull‐down experiments (Figure 8E). In addition, the combination of FGF13 and MTCH2 was significantly reduced in MPP^+^‐treated neurons, as demonstrated by mass spectrum and co‐immunoprecipitation (CO‐IP) assays (Figure 8A,F,G). *Mtch2* knockdown in primary neuron cultures partially canceled the effects of *Fgf13* overexpression on the release of stressful mitochondria (Figure S8C, Supporting Information; Figure 8H–K). Additionally, *Mtch2* interference abrogated the protective effects of *Fgf13* overexpression on neurons co‐cultured with mixed glia (Figure S8E–G, Supporting Information). Moreover, it also counteracted the alleviatory effects of *Fgf13* overexpression on neuronal loss and glial activation in the midbrain of PD‐like mice (Figure S8D,H–L, Supporting Information). Collectively, this evidence suggests that FGF13 physically interacts with mitochondria through the outer mitochondrial protein MTCH2, to restrain mitochondrial release, which underlies the protective effects of FGF13 against PD.

### 2.9 Abacavir Elevates Neuronal FGF13 to Reduce Neuroinflammation and Dopaminergic Neuron Loss in the PD Mouse Model

**Figure 9:**
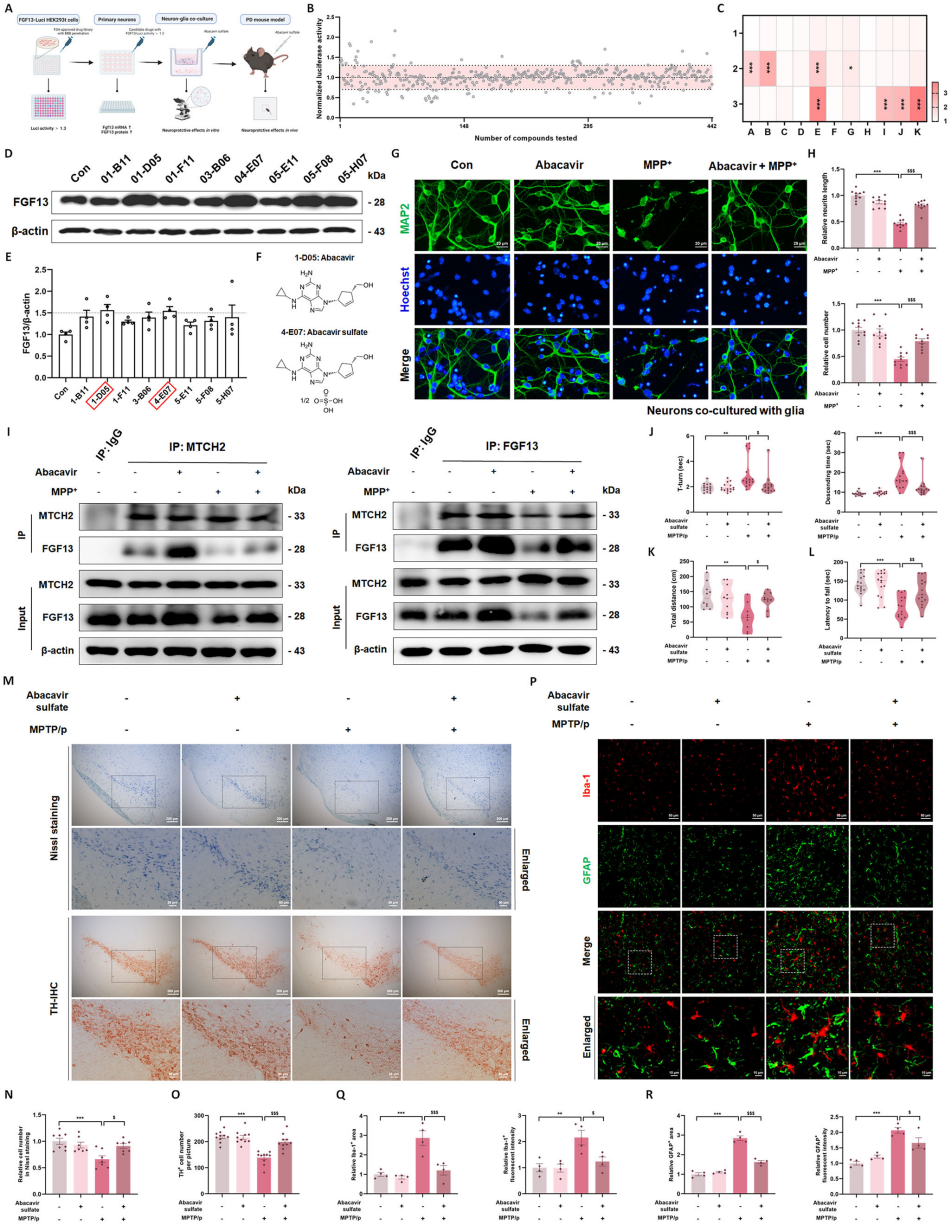
Abacavir elevates neuronal FGF13 to reduce neuroinflammation and dopaminergic neuron loss in the PD mouse model. A). Schematic diagram illustrating the process of screening for FGF13‐targeted neuroprotectants. B). Relative expression levels of *Fgf13* gene in HEK293T cells treated with a library of 442 FDA‐approved BBB‐penetrant drugs. C). *Fgf13* gene expression levels in primary neurons treated with candidate drugs detected by qRT‐PCR assay (*n* = 3 replicates per group; 1A: Control group, 2A: drug 1‐B11, 2B: drug 1‐D05, 2E: drug 1‐F11, 2G: drug 3‐B06, 3E: drug 4‐E07, 3I: drug 5‐E11, 3J: drug 5‐F08, 3K: drug 5‐H07). D). Representative immunoblots of FGF13 in primary neurons treated with candidate drugs. E). Quantitative analysis of FGF13 by immunoblotting (*n* = 4 replicate experiments per group). F). Structural formula of Abacavir and Abacavir sulfate. G). Representative fluorescent images of MAP2 (green) and Hoechst (blue) in primary neurons pretreated with Abacavir and then stimulated with MPP^+^. H). Relative neurite length and cell number in MAP2‐positive neurons (10 images per group from 3 independent experiments). I). Co‐immunoprecipitation assay of FGF13 and MTCH2 in primary neurons. J). Time for T‐turn and descending time in the pole test (*n* = 15 mice). K). Moving distance of mice in the OFT (*n* = 9–10 mice per group). L). Time of mice staying on the rotating rod in the rotarod test (*n* = 15 mice). M). Representative images of Nissl staining and representative immunohistological images of TH in the SNc of mice. N). Quantitative analysis of neuron numbers in the SNc of mice (*n* = 7–8 mice for Nissl staining). O). Quantitative analysis of TH‐positive neuron numbers in the SNc of mice (*n* = 10 mice per group). P). Representative fluorescent images of GFAP (green) and Iba‐1 (red) in the SNc. Q). Quantitative analysis of Iba‐1‐positive area and intensity in the SNc (*n* = 4 mice per group). R). Quantitative analysis of GFAP‐positive area and intensity in the SNc of mice (*n* = 4 mice per group). All data are presented as the mean ± s.e.m. ^*^ *p* < 0.05, ^**^ *p* < 0.01, and ^***^ *p* < 0.001 versus. Con or Saline group; $*p* < 0.05, $$*p* < 0.01, and $$$*p* < 0.001 versus. MPP^+^ or MPTP/p group. Statistical comparison was performed using one‐way ANOVA with Dunnett's multiple comparisons test.

After an in‐depth exploration of the molecular mechanisms underlying the correlation between FGF13 and PD, we hypothesize that potential chemicals targeting FGF13 may show efficacy in reversing PD progression. To this end, we constructed a HEK293T cell line using a luciferase reporter system, in which the *Fgf13* promoter region was linked to the luciferase coding sequence. We utilized this HEK293T cell line to screen for compounds that can upregulate FGF13 gene expression in a panel of 422 FDA‐approved drugs with blood‐brain barrier (BBB) penetration. The strategy for drug screening was depicted in **Figure** **9**A. Among 422 drugs, 32 drugs were identified to increase the *Fgf13* level by a ratio above 1.30 (Figure 9B). In the subsequent step, primary neurons were employed to investigate the effects of these drugs on *Fgf13* level by qRT‐PCR analysis. It was discovered that eight candidate drugs significantly increased *Fgf13* gene level (Figure 9C). Further immunoblotting analysis (Figure 9D,E) demonstrated that among the eight tested drugs, the top two compounds that significantly elevated FGF13 protein levels were candidate drug 1‐D05 (Abacavir) and drug 4‐E07 (Abacavir sulfate). Of note, the two drugs are essentially identical and are used for treating human immunodeficiency virus (HIV) infection in conjunction with other antiretroviral drugs. The chemical structures of the two compounds are shown in Figure 9F. Abacavir sulfate is the product ingredient and will dissociate to its free base Abacavir in vivo. We therefore administrated the mice with Abacavir sulfate and treated the primary cells with Abacavir in the following experiments. It was found that Abacavir treatment protected neurons co‐cultured with glia against MPP^+^‐induced cell damage (Figure 9G,H). Mechanistically, Abacavir promotes the interaction between FGF13 and MTCH2 (Figure 9I), thereby reducing the levels of stressful mitochondria in the neuronal culture medium (Figure S9A–D, Supporting Information). In the subsequent in vivo studies (flowchart shown in Figure S9E, Supporting Information), Abacavir sulfate increased FGF13 levels in the midbrain of mice (Figure S9F,G, Supporting Information) and alleviated the voluntary behavioral impairments induced by chronic MPTP administration. Specifically, it improved the decreased moving distance in the OFT, shortened the latency to fall from the rotating rod in the rotarod test, and reduced the T‐turn and descending times in the pole test (Figure 9J–L). Subsequently, loss of neurons detected by Nissl staining and the demise of TH‐positive neurons by IHC staining were remarkably reversed upon the administration of Abacavir sulfate (Figure 9M–O). Moreover, Abacavir sulfate pre‐administration inhibited the activation of astrocytes and microglia in nigrostriatal tissues of MPTP/p mice (Figure 9P–R; Figure S9H,I, Supporting Information). To confirm that the neuroprotective effects of Abacavir sulfate were FGF13‐dependent, we administered Abacavir sulfate to mice with *Fgf13* knockdown and then established the MPTP/p model. It was observed that *Fgf13* knockdown abolished the effects of Abacavir sulfate on MPTP/p‐induced neuron/dopaminergic neuron loss and glial reactivity (Figure S9J–M, Supporting Information). These findings demonstrate that the elevation of FGF13 mediated by Abacavir suppresses mitochondria‐derived damage signals and confers neuroprotection in the MPTP/p‐induced PD mouse model.

## 3 Discussion

**Figure 10:**
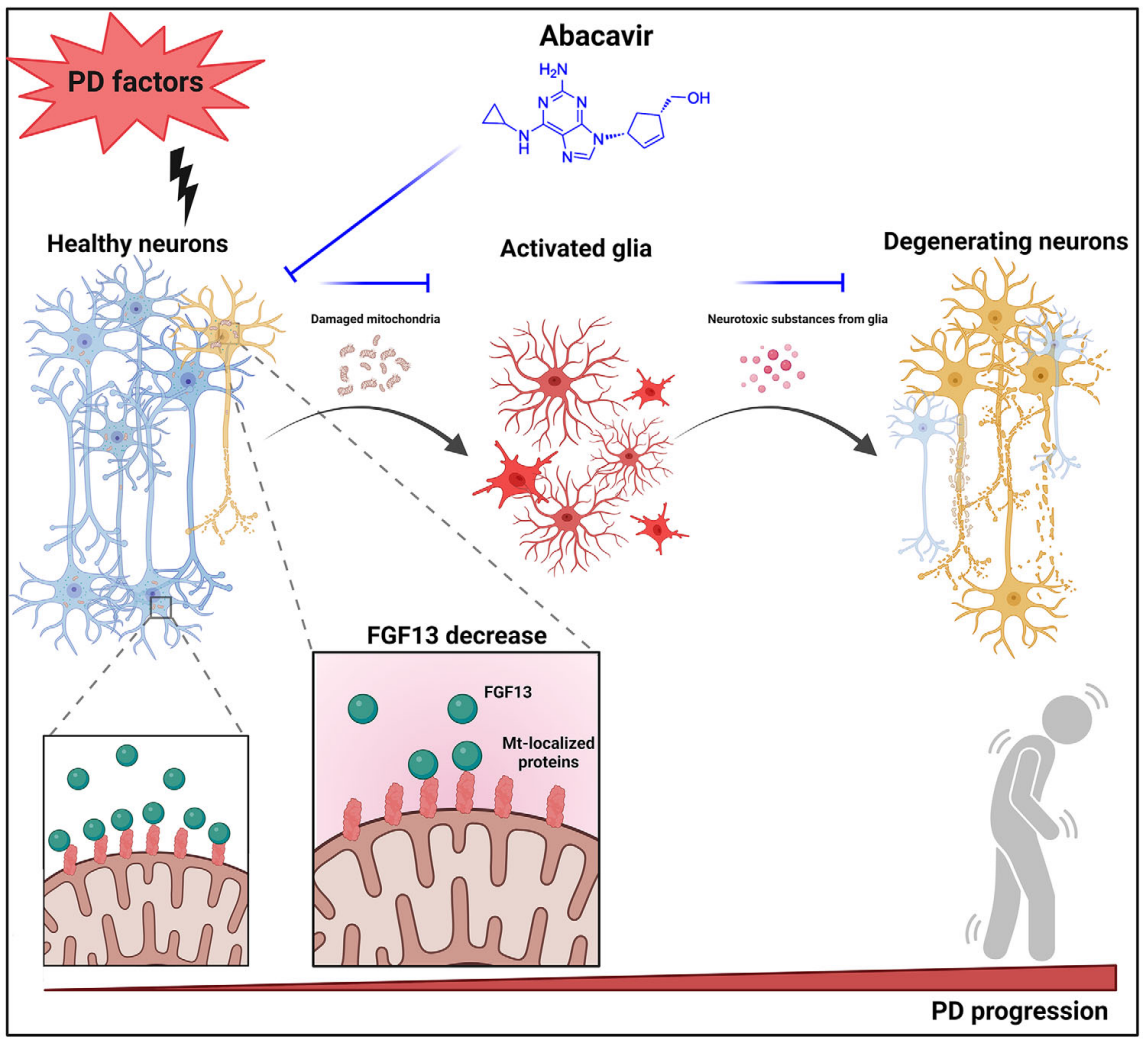
The schematic model of neuronal FGF13 in inhibiting mitochondria‐derived damage signals to prevent neuroinflammation and neurodegeneration.

In this study, we elucidate a novel role of FGF13 in mediating the degeneration of nigral neurons by modulating the release of neuronal mitochondria to neighboring glia. Specifically, FGF13 is identified as a mitochondria‐stabilizing protein that interacts with multiple mitochondrial proteins. Under PD‐associated stress, the reduction of neuronal FGF13 fails to retain mitochondria within the cytoplasm, leading to excessive release of stressed mitochondria. This release of mitochondria triggers glial reactivity, and in turn propagates neurodegeneration. Genetic upregulation or pharmacological elevation of FGF13 restrains mitochondrial efflux and inhibits neuroinflammation. Our findings suggest that FGF13 plays a previously unrecognized role in neuronal fate by mediating neuron‐glia interactions via mitochondrial transfer. Additionally, we provide the first evidence that Abacavir, a nucleoside reverse transcriptase inhibitor, can protect nigral dopaminergic neurons from damage, highlighting its potential as a therapeutic approach (graphic abstract shown in **Figure** **10**).

Selective neuronal and regional vulnerability is a critical feature for neurodegenerative diseases. For PD, dopaminergic neurons in the SNpc (A9 area) are the primary focus of the progressive neuronal loss.^[^ ^25^ ^]^ However, the mechanisms underpinning their selective vulnerability in PD have been challenging to elucidate. We aimed to utilize tissue‐specific expression databases from patient samples to characterize the properties of vulnerable neurons. After analyzing the PD expression profile data for human SN tissue, we identified 21 overlapping genes that exhibited consistent changes across multiple GEO datasets. Among these, several genes encode proteins proteins critical for dopamine metabolism, transport, and dopamine signal transduction including *TH*, *DDC*, *SLC18A2*, *SLC6A3*, *ALDH1A1*, and *KCNJ6*.^[^ ^26^, ^27^, ^28^ ^]^ Additionally, we identified genes essential for the development of the mesencephalic dopaminergic system such as *DLK1* and *RET*.^[^ ^29^, ^30^ ^]^ Most of these genes are used as histochemical markers to label dopaminergic neurons and their subtypes.^[^ ^31^, ^32^, ^33^ ^]^ Notably, *AGTR1* is found to localize in ventral tier of SNpc and marks one population of dopaminergic neurons that are highly susceptible to loss in PD.^[^ ^34^ ^]^ *SV2C*, involved in synaptic function, has been evaluated as a functional PD candidate gene due to its restricted expression in brain regions associated with PD, its role in mediating dopamine homeostasis, and its role as novel asian‐specific PD risk loci.^[^ ^35^, ^36^ ^]^ *ANK1* exhibits brain region‐ and disease‐specific differential DNA methylation in multiple neurodegenerative diseases.^[^ ^37^ ^]^ *CADPS2*, which mediates synaptic vesicle dynamics, is transcriptionally decreased in PD patients harboring *LRRK2* mutations but highly expressed in degenerating dopaminergic neurons in idiopathic PD.^[^ ^38^, ^39^ ^]^ The involvement of other genes in PD remains to be clarified.

As previously reported, mutations in FHFs often lead to neurological disorders.^[^ ^17^, ^18^ ^]^ Among the four FHFs members, *Fgf13* has been identified as a candidate gene of X‐chromosome‐linked mental retardation based on human genetic studies.^[^ ^19^ ^]^ In mice, *Fgf13* deficiency causes weakened learning and memory.^[^ ^22^ ^]^ Regarding motor abilities, whole‐body *Fgf13* knockout mice exhibit normal behavior.^[^ ^22^ ^]^However, cell‐specific deletions reveal distinct phenotypes: *Fgf13* deletion in the cerebral cortex leads to a slight increase in spontaneous movement,^[^ ^22^ ^]^ while deletion in neural stem cells (NSCs) leads to hyperactivity.^[^ ^23^ ^]^ In our current study, we found no significant changes of voluntary motor abilities in mice with neuron‐specific *Fgf13* overexpression in the SN. These findings underscore the cell‐specific and region‐specific roles of FGF13 in brain function. Indeed, the cell‐specific effects of FGF13 are also evident in psychiatric phenotypes. Global *Fgf13* knockout reduces depression‐like behaviors, whereas *Fgf13* depletion in NSCs induces anxiety‐like behaviors.^[^ ^22^, ^23^ ^]^ Therefore, the complex roles of FGF13 in neurological processes warrant further investigation. The *Fgf13* gene harbors distinct isoforms including FGF13A, which contains a nuclear localization sequence, and FGF13B, which is primarily cytoplasmic.^[^ ^23^, ^40^ ^]^ Our study confirmed that neuronal FGF13 is expressed in both the nucleus and cytoplasm, while astrocytic FGF13 is predominantly nuclear. These results align with previous findings that neurons express both FGF13A and FGF13B, whereas astrocytes primarily express FGF13A.^[^ ^23^ ^]^ Although we do not specifically differentiate the effects of FGF13A or FGF13B in the current study, we speculate that the cytoplasmic FGF13B isoform plays a critical role in PD by stabilizing mitochondria within the cytoplasm.

Mitochondria are not only inherited vertically through cell division but can also be transferred horizontally between cells.^[^ ^20^ ^]^ Once released outside the cell, mitochondria often function as signaling organelles that facilitate cell‐to‐cell communication. Multiple models of mitochondria‐dependent cell interplay have been reported in the brain, with the majority focusing on the neuroprotective roles of healthy or functional mitochondria.^[^ ^10^, ^11^ ^]^ However, mitochondria can also act as auto‐pathogens when released from cells, triggering immune responses and inflammation due to their structural and molecular similarities to bacteria.^[^ ^21^ ^]^ In this study, we validated the existence of damaged mitochondria in the medium of primary neurons under stress conditions. We further demonstrated that these abnormal mitochondria can induce inflammatory responses in neighboring microglia and astrocytes. Although we are not focusing on the downstream events of glia‐mediated neuronal injury, we propose that activated glia, stimulated by neuronal mitochondria, may release their own stressed mitochondria to transmit and amplify the degenerative signals in a vicious cycle, as previously reported.^[^ ^8^ ^]^

Members of the FHF family are devoid of the typical signal peptides for secretion.^[^ ^14^ ^]^ However, they can still be secreted into the extracellular space, particularly under stress conditions.^[^ ^15^, ^16^ ^]^ In our research, the supplementation of FGF13 protein failed to reverse the neuronal damage induced by PD neurotoxins. As a result, FGF13 does not exert its neuroprotective effects through the membranous receptor‐binding mechanism in the current study. Instead, we have clarified that FGF13 binds to multiple mitochondrial proteins intracellularly. This binding anchors mildly stressed mitochondria, thereby preventing neuroinflammation. Combined with previous studies that have demonstrated FGF13 to be a microtubule‐stabilizing protein due to its ability to interact with microtubules,^[^ ^22^ ^]^ we propose that FGF13 serves as a bridging protein to tether mitochondria to the cellular microtubule network by interacting with both microtubules and mitochondrial proteins. This anchoring mechanism enables FGF13 to act as a mitochondrial stabilizer, maintaining the stable position of mitochondria within the cell and preventing their release into the extracellular space. Notably, although FGF13 can interact with multiple mitochondrial proteins, overexpression of *Fgf13* scarcely affects the mitochondrial mass or functions in neurons treated with PD toxin. We ascribe this to the diverse and counterbalancing roles of these binding proteins in stressed neurons. Perhaps more importantly, glial cells respond more sensitively and effectively to extracellular signals than neurons do.

Among the mitochondrial proteins that bind to FGF13, MTCH2 is a surface‐exposed outer mitochondrial membrane protein and plays a crucial role in maintaining mitochondrial functionality. It regulates mitochondrial death pathway,^[^ ^41^, ^42^ ^]^ mitochondrial oxidative phosphorylation,^[^ ^43^, ^44^ ^]^ and mitochondrial outer membrane protein biogenesis.^[^ ^24^ ^]^ These mitochondrial process may provide functional basis for mitochondrial transfer. Furthermore, MTCH2 has been reported to affect mitochondrial motility and distribution,^[^ ^45^ ^]^ potentially providing the necessary driving force for cell‐to‐cell mitochondrial transfer. Additionally, MTCH2 plays crucial roles in maintaining mitochondrial structures via fusion and fission processes,^[^ ^44^, ^46^, ^47^ ^]^ which are critical aspects of mitochondrial dynamics and significantly influence mitochondrial transfer. Specifically, MTCH2's involvement in mitochondrial fusion and fission could influence the formation and release of mitochondria‐derived vesicles or the transfer of free mitochondria between cells.

Dopamine replacement therapy remains a cornerstone of PD treatment due to its efficacy in alleviating motor symptoms. However, their limitations including sever side effects and lack of long‐term efficacy are significant concerns. There is an urgent need for disease‐modifying therapies that can prevent the progression of refractory PD.^[^ ^2^ ^]^ Recently, the repurposing of approved drugs has emerged as an attractive strategy to accelerate the development of new neuroprotective agents.^[^ ^48^, ^49^ ^]^ In this context, we screened FDA‐approved drugs to identify compounds targeting FGF13 for their potential therapeutic effects in PD. Our study identified Abacavir, a nucleoside reverse transcriptase inhibitor primarily used in antiretroviral therapy, as a promising neuroprotective agent. Although Abacavir has rarely been reported in the context of brain diseases, several lines of evidence support its potential neuroprotective effects. It is demonstrated that activation of human endogenous retroviruses contributes to neurodegenerative processes of motor neuron disease.^[^ ^50^ ^]^ Furthermore, Abacavir has been reported to attenuate the senescent and inflammatory phenotypes in long‐term neuronal cultures, potentially delaying brain aging.^[^ ^51^ ^]^

Altogether, our study highlights for the first time that FGF13 protects neurons against degeneration by restraining the transfer of stressful mitochondrial to adjacent glia, and thereby preventing irrepressible neuroinflammation. Of particular significance, Abacavir, which targets the elevation of neuronal FGF13, effectively combats mitochondria‐derived damage signals and demonstrates efficacy in relieving neurodegenerative phenotype in a mouse model for PD.

## 4 Experimental Section

### Animals

All animal‐related experiments were carried out in strict compliance with the institutional ethical guidelines for animal care at Nanjing Medical University (IACUC – 2008067). The mice were housed in a specific pathogen‐free (SPF) environment. The room was maintained at a constant ambient temperature of 22 ± 2 °C and operated on a 12‐h light/dark cycle. The animals had ad libitum access to sterilized SPF pellet rodent feed and sterilized water. In the present study, 3‐month‐old male C57BL/6J mice were utilized. These mice were supplied by the Comparative Medicine Centre of Yangzhou University.

### Mice Model Establishment and Drug Administration

For the establishment of different mouse models for PD, mice were divided into six groups. For the subacute MPTP model,^[^ ^52^, ^53^, ^54^ ^]^ MPTP (Sigma–Aldrich, Cat#M0896; 20 mg·kg^−1^ body weight, in saline, s.c.) was administered at 24 h intervals for five consecutive days. Control mice were treated with saline. For chronic MPTP (MPTP/p) model,^[^ ^55^, ^56^ ^]^ MPTP (20 mg·kg^−1^ body weight, in saline, s.c.) plus a clearance inhibitor probenecid (MedChemExpress, Cat#HY‐B0545; 250 mg·kg^−1^ body weight, in dimethyl sulphoxide, i.p.) was administered every 3.5 days for five weeks. Probenecid was injected subcutaneously at 1 h intervals after MPTP administration. Control mice were treated with saline and probenecid. For PFF‐induced mouse model, 1 µg α‐syn PFF was micro‐injected into the striatum. Control mice were received phosphate‐buffered saline (PBS) micro‐injection. Six month later, PFF mice were sacrificed for bioassay.

For the administration of Abacavir sulfate, mice were injected everyday with Abacavir sulfate (Aladdin, Cat#A129792; 125 mg·kg^−1^ body weight, in sterile water, i.g.) from three days before MPTP/p administration to the last day of PD model establishment. The dose of Abacavir sulfate administered to mice was determined based on the equation: animal equivalent dose (mg·kg^−1^) = Human dose (mg·kg^−1^) * Km ratio (the human dose of abacavir sulfate is 600 mg · 60 kg^−1^ and the Km ratio is 12.3.), as previously reported.^[^ ^57^ ^]^ Control mice were treated with sterile water.

### Stereotaxic Micro‐Injection

The animals were securely positioned on a stereotaxic apparatus and anesthetized with 2% isoflurane (2 L·min^−1^ oxygen flow rate). An incision was made in the skin to reveal the bregma point and the area around the incision was trimmed and disinfected. For PFF micro‐injection into the striatum, 1 µL of α‐syn PFF at a concentration of 5 µg·µL^−1^ was injected into the striatum with the following injection coordinates relative to bregma: anterior posterior (AP), +0.86 mm; medial lateral (ML), ±1.5 mm; dorso ventral (DV), −3.0 mm. 1 µL PBS was injected into the same position of control mice. For the micro‐injection of AAV, AAV vectors were utilized packaged with the *Fgf13* plasmid, *Fgf13* shRNA, or *Mtch2* shRNA. Specifically, the vectors were rAAV‐hSyn‐*Fgf13*‐EGFP‐WPRE‐hGH polyA (serotype 2/9, 5×10^12^ vg mL^−1^), rAAV‐hSyn‐*Fgf13* shRNA‐EGFP‐WPRE‐hGH polyA (serotype 2/9, 5×10^12^ vg mL^−1^) and rAAV‐hSyn‐*Mtch2* shRNA‐WPRE‐hGH polyA (serotype 2/9, 1×10^12^ vg mL^−1^). The shRNA sequences are listed in Table S2 (Supporting Information). Additionally, HBAAV‐Syn‐Mito‐dsRed (serotype 2/9, 1×10^12^ vg mL^−1^) was used for neuronal mitochondrial labeling. Anaesthetized mice were injected with 1.0 µL AAV at the following coordinates relative to bregma: AP, −3.0 mm; ML, ±1.3 mm; DV, −4.5 mm. One microliter of control AAV was injected into the same position of control mice. A 10 µL Hamilton syringe was used to inject the PFF or AAV at a rate of 0.2 µL· min^−1^. To prevent liquid backflow, the needle was kept in place for 5 min and then slowly withdrawn. The skin was sutured and disinfected. After the surgery, mice were subjected to standard rearing conditions for subsequent assay. Three weeks after injection, viral expression was confirmed by detecting EGFP or dsRed fluorescence in midbrain slices.

### Behavioral Analysis

Behavioral analysis were performed as described previously.^[^ ^52^ ^]^ The pole test, rotarod test and open field test were performed at 3 days after the final injection of MPTP. Open field test was used to detect the locomotor activity of mice. Specifically, the mouse was placed into activity monitor chambers (20 cm × 20 cm × 15 cm) for 30 min, and the activities were recorded at 5‐min intervals. For the pole test, the mice were placed head upward on the top of a vertical wooden rough‐surfaced pole (diameter 1 cm, height 50 cm). The time needed for the mouse to turn completely head downward was recorded (T‐turn). Total time until the mouse reached the floor with its four paws was recorded (descending time). For the rotarod test, mice were trained two times in the apparatus with an accelerated speed from 5 to 20 rpm over 5 min. In the formal experiment, mice were placed on the rotating rod and tested at 20 rpm for 180 s. Time that each mouse stayed on the rotating rod (latency to fall) was recorded.

### Primary Cell Cultures and Treatments

Primary cell cultures were conducted as described previously with some modifications.^[^ ^52^, ^58^ ^]^ For primary neuron cultures or primary dopaminergic neurons cultures, brain or mesencephalon was isolated from embryos (embryonic day 15–16) of C57BL/6J mouse strain. Tissues removing the meninges microscopically were digested with 0.025% trypsin (Gibco, Cat#27250018) at 37 °C for 30 min, followed by termination with Dulbecco's modified Eagle's medium (DMEM, Gibco, Cat#12100‐046) supplemented with 10% fetal bovine serum (FBS, Sigma–Aldrich, Cat#F8318). The suspension was filtered with a 40 µm filter (BD Falcon, Cat#352340) and centrifuged at 1000 g for 5 min. The cell precipitate was re‐suspended in Neurobasal medium (Gibco, Cat#21103049) supplemented with 2% B‐27 (Gibco, Cat#17504044) and GlutaMAX (Gibco, Cat#35050079), and then the cells were seeded on plates pre‐coated with 0.1 mg·mL^−1^ Poly‐D‐lysine (Gibco, Cat#A3890401). Neurons were cultured for 7 days, and the medium was replaced with fresh medium every 3.5 days. For primary microglia and astrocyte cultures, the brain tissues of neonatal mice aged 1–3 day were stripped of meninges and blood vessels under a microscope. Then the tissues were digested with 0.25% trypsin for 2 min and terminated by DMEM supplemented with 10% FBS. Cell suspension was filtered with a 40 µm filter and centrifuged at 1000 g for 5 min. Cells were re‐suspended in DMEM/F‐12 (Gibco, Cat#11320033) supplemented with 10% FBS and 1% penicillin/streptomycin (P/S, Gibco, Cat#15640055) and then plated in T‐75 flasks (Corning, Cat#CLS430641). The cell medium was replaced with fresh medium 24 h later and then refreshed every 3 days. After 2 weeks in culture, primary microglia were isolated by gently shaking the flask and then detached microglia were plated for the subsequent experiments. After removal of microglia 2–3 times, the astrocytes were split onto culture plates for the subsequent experiments. Cells were cultured at 37 °C in a 5% (v:v) CO_2_ atmosphere. For mixed glia cultures, primary astrocytes were first seeded onto culture plates for 3 days and then the detached microglia were plated for the subsequent experiments.

The primary neurons were directly exposed to MPP^+^ at concentrations of 1, 10, and 100 µm respectively for 24 h. For the treatment of both primary neurons and primary neurons co‐cultured with glia, 10 µm MPP^+^ was finally used to stimulate the cells for 24 h. Specifically, neurons were first transfected with LV packaged with *Fgf13* plasmid, *Fgf13* shRNA or a mixture of LV packaged with *Fgf13* plasmid and LV packaged with *Mtch2* shRNA (sequences were listed in Table S2, Supporting Information), and then co‐cultured with glia followed by the treatment of MPP^+^. Otherwise, the neurons were primed with Abacavir (MedChemExpress, Cat#HY‐17423; 1 µm, 1 h), and then co‐cultured with glia followed by the treatment of MPP^+^. For the treatment of mixed glial cell cultures in each well, the free mitochondria that were isolated from the neuronal medium in the corresponding well were used to incubate the cells for 24 h. During this incubation, glial cells in the groups receiving mitochondria from MPP^+^‐treated neurons were also treated with 10 µm MPP^+^ to mimic the in vivo environment.

### Immunohistochemistry and Immunocytochemistry

For immunohistochemistry (IHC) of brain slices, the brain tissues were dehydrated with 20% and then 30% sucrose dissolved in PBS for three days respectively after post‐fixation in 4% paraformaldehyde (PFA). Then the brains embedded in OCT were cut into 20 µm‐thick slices on coronal plane. Brain slices were simply washed three times in PBS. All slices were subsequently blocked with 5% BSA and 0.3% Triton X‐100 in PBS (5% BSA/PBST), and then incubated with primary antibody in 4 °C overnight followed by incubation of the secondary antibody at room temperature for 1 h. In Diaminobenzidin (DAB)‐dependent IHC analysis, brain sections are incubated with 3% hydrogen peroxide to quench the endogenous peroxidase activity before blocking with 5% BSA/PBST. After rinsing with PBS following horseradish peroxidase (HRP)‐labeled secondary antibody incubation, the slices were visualized by the DAB (Boster, Cat#AR1002) reaction for 5 min. IHC images were visualized under the microscope (Olympus) with stereo Investigator software, which was used to count the number of positive cells. Fluorescently labeled sections were visualized with the confocal scanning laser microscope (Carl Zeiss). The fluorescent area in each image was quantified in ImageJ software. For immunocytochemical staining, primary cells were rinsed with PBS and then fixed with 4% PFA for 20 min. Cell slides were then followed the same procedures as immunohistochemistry of brain slices.

The primary antibodies used were as follows: mouse anti‐FGF13 antibody (1:300, Invitrogen, Cat#MA5‐27705), rabbit anti‐TH antibody (1:800, Proteintech, Cat#25859‐1‐AP), rabbit anti‐MAP2 antibody (1:500, Proteintech, Cat#17490‐1‐AP), rabbit anti‐GFAP antibody (1:500, Cell Signaling Technology, Cat#80788), rabbit anti‐Iba‐1 antibody (1:500, Asis biofarm, Cat#OB‐PRB029), mouse anti‐GFAP antibody (1:500, Cell Signaling Technology, Cat#3670), mouse anti‐CD44 antibody (1:200, Proteintech, Cat#60224‐1‐Ig), mouse anti‐CD68 antibody (1:50, Santa Cruz Biotechnology, Cat#SC‐52998), rabbit anti‐C16 antibody (1:200, Abcam, Cat#ab246222), goat anti‐Iba‐1 antibody (1:500, Abcam, Cat#ab5076), rabbit anti‐MTCH2 antibody (1:200, Proteintech, Cat#16888‐1‐AP). The fluorescent secondary antibodies were as follows: Alexa Fluor™ 488 Goat anti‐Rabbit IgG antibody (1:1000, Invitrogen, Cat#A32731), Alexa Fluor™ 555 Goat anti‐Rabbit IgG antibody (1:1000, Invitrogen, Cat#A21428), Alexa Fluor™ 647 Goat anti‐Rabbit IgG antibody (1:1000, Invitrogen, Cat#A32731), Alexa Fluor™ 488 Goat anti‐Mouse IgG antibody (1:1000, Invitrogen, Cat#A11001), Alexa Fluor™ 555 Goat anti‐Mouse IgG antibody (1:1000, Invitrogen, Cat#A21422), Alexa Fluor™ 647 Goat anti‐Mouse IgG antibody (1:1000, Invitrogen, Cat#A21235) and Alexa Fluor® 555 Donkey anti‐Goat IgG antibody (1:1000, Invitrogen, Cat#A21432).

### Nissl Staining

For Nissl staining, the brain slices were mounted onto the slides to dry naturally. The slides were soaked in the mixture of solution A and B from nissl staining kit (KeyGEN, Cat#KGA4104) as the manufacture's instruction, and then dehydrated with alcohol and xylene. The images were visualized under the microscope (Olympus), and the stereo Investigator software was used to count the number of positive cells.

### Protein extraction and Immunoblots

Cells and brain tissues were lysed in lysis buffer containing protease inhibitor (Thermo Fisher, Cat#A32961). After centrifugation, protein concentration of the lysate surpernatant was measured with BCA Protein Assay Kit (Thermo Fisher Scientific, Cat#23250) and equal amount of protein was separated by sodium dodecyl sulphate‐polyacrylamide gel electrophoresis (SDS‐PAGE) electrophoresis. Proteins were then transferred from gel to 0.20 µm nitrocellulose membrane (Pall Corporation, Cat#66485). Membranes were blocked with 5% non‐fat milk in Tris‐buffered saline with Tween‐20 (TBST) for 1 h, following by incubation with primary antibodies at 4 °C overnight. The following primary antibodies were used: mouse anti‐FGF13 antibody (1:1000, Invitrogen, Cat#MA5‐27705), rabbit anti‐TH antibody (1:1000, Proteintech, Cat#25859‐1‐AP), mouse anti‐NLRP3 antibody (1:1000, AdipoGen, Cat#AG‐20B‐0014‐C100), rabbit anti‐NF‐κB (P65) antibody (1:1000, Cell Signaling Technology, Cat#8242), rabbit Phospho‐NF‐κB (P65) antibody (1:1000, Cell Signaling Technology, Cat#3033), rabbit anti‐Caspase‐1 antibody (1:1000, Millpore, Cat#06‐503‐1), goat anti‐IL‐1β antibody (1:1000, Sigma–Aldrich, Cat#I3763), rabbit anti‐Histone H3 antibody (1:2000, Proteintech, Cat#17168‐1‐AP), mouse anti‐β‐actin (1:2000, Proteintech, Cat#66009‐1‐Ig). HRP‐conjugated Goat Anti‐Mouse IgG (H+L) (1:2000, Proteintech, Cat#SA00001‐1) and HRP‐conjugated Goat Anti‐Rabbit IgG (H+L) (1:2000, Proteintech, Cat#SA00001‐2) secondary antibodies were used at dilution of 1:5000. The bands were detected by ImageQuant LAS 4000 imaging system (GE Healthcare). For densitometric analyses, immunoreactive bands were quantified using ImageJ software.

### RNA Isolation and qRT‐PCR

Total RNA was extracted from mouse tissues or primary cells using TRIzol reagent (Invitrogen, Cat#15596026) and then reversely transcribed with the ChamQ Universal SYBR qPCR Master Mix (Vazyme, Cat#Q711). The cDNAs obtained were mixed with FastStart Universal SYBR Green Master (Vazyme, Cat#R312) and gene‐specific primers (Table S1, Supporting Information) for qRT‐PCR in a StepOnePlus instrument (Applied Biosystems, USA). GAPDH served as an internal control.

### GST Pull‐Down Assay

The recombinant GST‐tagged MTCH2 (GST‐MTCH2) fusion protein was expressed in *Escherichia coli* and then purified using glutathione‐agarose beads from the GST Protein Interaction Pull‐Down Kit (Pierce, Cat#21516). Similarly, the His‐FGF13 fusion protein was also expressed and purified. Specifically, the glutathione‐agarose beads coated with GST‐MTCH2 were incubated with His‐FGF13 in the binding buffer at 4 °C for 12 h under gentle rotation. After a thorough washing step, the bound proteins were eluted and subsequently analyzed via western blotting with the utilization of anti‐His and anti‐GST antibodies.

### Co‐IP Assay

Cell samples were lysed using a buffer containing protease inhibitors. Equal amounts of protein were subsequently incubated with anti‐FGF13 or anti‐MTCH2 antibodies at 4 °C overnight. Protein A/G PLUS‐Agarose (Santa Cruz Biotechnology, Cat#SC‐2003) was added and incubated with samples for 4 h at room temperature. The IP complexes were washed three times with lysis buffer, denatured by the addition of 2.5 × loading buffer, and boiled for 5 min. The IP complexes were further analyzed by western blotting.

### Label‐Free Mass Spectrometry

Anti‐FGF13 immunoprecipitated proteins (500 µg per sample) were digested following the filter‐aided sample preparation procedure. After reduction and alkylation, an appropriate amount of enzyme (mass ratio of 1:50) was added to the test sample and subjected to enzymatic hydrolysis at 37 °C for 20 h. The resulting peptides were desalted, freeze‐dried, and then redissolved in a 0.1% formic acid solution. MS experiments were performed on a Q Exactive mass spectrometer coupled to an Easy nLC system. Five micrograms of the peptide were loaded onto a C18‐reversed‐phase column in buffer A (0.1% formic acid) and separated with a linear gradient of buffer B (0.1% formic acid in 84% acetonitrile) at a flow rate of 250 nL·min^−1^ controlled by IntelliFlow technology over 120 min. MS data were acquired using a data‐dependent top10 method, which dynamically chooses the most abundant precursor ions from the survey scan (m/z 300–1800) for HCD fragmentation. Determination of the target value is based on predictive automatic gain control. Survey scans were acquired at a resolution of 70,000 at m/z 200, and the resolution for HCD spectra was set to 17,500 at m/z 200. The instrument was run with peptide recognition mode enabled. MS experiments were performed in triplicate for each sample. The MS data were processed using ProteomeDiscoverer 2.5 and searched against the UniProtKB database for protein identification and quantification.

### High‐Performance Liquid Chromatography

Striatum tissue samples were homogenized with buffer containing 0.1 M HClO_4_ and 0.1 mm EDTA, then were centrifuged (20,000g, 4 °C) for 30 min. The supernatant was collected and then injected into the mobile phase (90 mm sodium phosphate monobasic, 50 mm citrate, 1.7 mm 1‐octane sulfonic acid, 50 µm EDTA, 10% acetonitrile) with the flow rate of 0.2 mL·min^−1^. Dopamine and dihydroxyphenyl acetic acid (DOPAC) were detected by ESA Coulochem III electrochemical detector (Thermo Fisher Scientific, USA). Samples were quantified by internal standard method comparing with the standard (Sigma–Aldrich, USA).

### ELISA Assay

To determine the levels of inflammatroy cytokines and chemokines in the brain lysates, protein concentration of the lysates was conducted and each sample should be adjusted to the same concentration. Then the lysates were analyzed using commercial ELISA kits following the manufacturer's instructions. Cell medium of primary cell cultures was collected and centrifuged to remove cell debris. Then the medium was analyzed using commercial ELISA kits following the manufacturer's instructions.

### Isolation of Extracellular Mitochondria

For isolation of extracellular mitochondria, the supernatant of primary neuron cultures was centrifuged at 1,000 g for 10 min to pellet cellular debris. Following this step, the supernatant was centrifuged at 13,000 g for 30 min to collect the mitochondria‐containing pellet. Mitochondria were visualized by the mitochondrial fluorescent probe Mitotracker green (Invitrogen, Cat#M46750) and transmission electron microscopy.

### Flow cytometry Assay

Isolated mitochondria were stained with fluorescent dyes and then analyzed by flow cytometry (FACS Calibur, BD, USA). At least 5000 events per sample were collected for data analysis. Mitotracker green (Invitrogen, Cat#M46750) was used for staining mitochondria, and MitoSOX Red (Invitrogen, Cat#M36008) was to measure mitochondrial superoxide production. The change of mitochondrial membrane potential was measured as the ratio between aggregate and monomeric forms of JC‐1 (Invitrogen, Cat#T3168).

### Transmission Electron Microscopy

The extracellular mitochondria‐containing pellets were collected and then transferred to 3% glutaraldehyde for further processing at 4 °C. Then the samples were post‐fixed in 1% osmium tetroxide, dehydrated in a series of acetone solutions, and embedded in Epox 812. Semithin sections were stained with methylene blue, and ultrathin sections were cut using a diamond knife, stained with uranyl acetate and lead citrate.

### Construction of Stable FGF13‐Luciferase Cell Line

To construct a stable HEK293T cell line expressing an FGF13 luciferase reporter, the promoter region (1300 bp upstream of the 5'UTR) from the MANE‐recommended FGF13 transcript (transcript variant 1) was cloned into the pLV2ltr‐PGK‐Puro‐(ΔPro)‐Luc vector. The construct was validated by sequencing to ensure accurate cloning and then extracted using an endotoxin‐free plasmid kit. After confirming puromycin sensitivity (0.5 µg mL^−1^), lentivirus was packaged with the plasmids and used to infect cells. Infected cells were selected with puromycin, and monoclonal lines were established by limited dilution. The presence of the FGF13 promoter‐luciferase construct was confirmed by PCR and sequencing.

### Statistical Analysis

Statistical significance was determined using GraphPad Prism 8. Unpaired two‐tailed Student's t‐test, one‐way ANOVA, two‐way ANOVA or Pearson correlation test was conducted according to test requirements. ^*^ *p* < 0.05, ^**^ *p *< 0.01, and ^***^ *p* < 0.001 were considered significant. The number of replicates, repeats of individual experiments and statistical tests are indicated in the legends.

## Conflict of Interest

The authors declare no conflict of interest.

## Author Contributions

M.L. and G.H. conceived and supervised the project, and made revisions to the manuscript. N.S. designed the experiments, wrote the paper and analyzed the data. N.S., X.W., and L.H. performed cell cultures, stereotaxic micro‐injection, behavioral tests, immunostaining, flow cytometry, western blotting, and qRT‐PCR with the help of L.H., S.M., Y.Z., and X.Y. N.S., and S.M. performed the bioinformatics analysis of the datasets. Y.L., Q.Z., and Y.Z. provide experimental guide. J.D. provided reagent and technical support. Y.L., Q.Z., and F.H. provided substantial input on the English writing of the manuscript, offering critical insights and suggestions that enhanced the overall quality of the work. All authors have reviewed the manuscript.

## Supporting information



Supporting Information

## Data Availability

The data that support the findings of this study are available from the corresponding author upon reasonable request.
