# Supplementary material for: Neuronal FGF13 Inhibits Mitochondria‐Derived Damage Signals to Prevent Neuroinflammation and Neurodegeneration in a Mouse Model of Parkinson's Disease
Source: Adv Sci (Weinh). 2025 May 8;12(28):2503579. doi: 10.1002/advs.202503579 (PMC12302648; doi:10.1002/advs.202503579)
Supplement: Supplementary file 1 — Supporting Information [file ADVS-12-2503579-s001.docx]

**Supplementary Materials**

**Neuronal FGF13 Inhibits Mitochondria-derived Damage Signals to Prevent Neuroinflammation and Neurodegeneration in a Mouse Model of Parkinson’s Disease**

Nanshan Song^1 *^, Xiangxu Wang^2^, Luqing Ha^3^, Lamei Hu^2^, Shuyuan Mei^4^, Yue Liang^2^, Yujie Zhao^2^, Xingyin Yang^4^, Qingyu Zhang^1^, Yuanzhang Zhou^1^, Jianhua Ding^2^, Yan Liu^2^, Qigang Zhou^2^, Feng Han^2^, Gang Hu^1,2,3 *^ and Ming Lu^2,5 *^

*^1^ School of Medicine, Nanjing University of Chinese Medicine, Nanjing 210023, China*

*^2^ Jiangsu Key Laboratory of Neurodegeneration, Department of Pharmacology, Nanjing Medical University, Nanjing 211166, China*

*^3^ School of Pharmacy, Nanjing University of Chinese Medicine, Nanjing 210023, China*

*^4^ The Clinical Medical College, Nanjing Medical University, Nanjing 211166, China*

*^5^ Changzhou Second People's Hospital, Changzhou Medical Center, Nanjing Medical University, Changzhou 213000, China*

*Address corresponding to:

Ming Lu, M.D., Ph.D.

Jiangsu Key Laboratory of Neurodegeneration

Department of Pharmacology, School of Basic Medical Sciences

Nanjing Medical University

101 Longmian Avenue, Nanjing, Jiangsu 211166, China

Tel: 86-25-86869339

Email: [lum@njmu.edu.cn](mailto:lum@njmu.edu.cn)

Gang Hu, M.D., Ph.D.

Jiangsu Key Laboratory of Neurodegeneration

Department of Pharmacology, School of Basic Medical Sciences

Nanjing Medical University

101 Longmian Avenue, Nanjing, Jiangsu 211166, China

Tel: 86-25-86863108

Email: [ghu@njmu.edu.cn](mailto:ghu@njmu.edu.cn)

Nanshan Song, M.D., Ph.D.

Department of Pharmacology, School of Medicine,

Nanjing University of Chinese Medicine

138 Xianlin Avenue, Nanjing, Jiangsu 210023, China

Email: [nssong@njucm.edu.cn](mailto:ghu@njmu.edu.cn)

1. **Supplementary Materials and Methods**

**1.1 Treatment of mice**

Mice were micro-injected with recombined FGF13 protein into the bilateral SN (substantia nigra) and then acute 1-methyl-4-phenyl-1,2,3,6-tetrahydropyridine (MPTP) model were established. MPTP (20 mg·kg^-1^ body weight, in saline, i.p.) was administered every 2 h for a total of four injections within one day. Control mice were treated with saline.

For the administration of Abcavir sulfate, mice were injected everyday with Abacavir sulfate (Aladdin, Cat#A129792; 125 mg·kg^-1^ body weight, in sterile water, i.g.) from three days before chronic MPTP administration to the last day of PD model establishment. For chronic MPTP (MPTP/p)-induced PD model, MPTP (20 mg·kg^-1^ body weight, in saline, i.p.) was administered every 3.5 days for five weeks. Probenecid (MedChemExpress, Cat#HY-B0545; 250 mg·kg^-1^ body weight, in dimethyl sulphoxide, i.p.) was administered every 3.5 days for five weeks at 1 h intervals after MPTP administration. Control mice were treated with saline and probenecid.

For the micro-injection of adeno-associated virus (AAV), including a mixture of AAV-*Fgf13* and AAV-*Mtch2* shRNA or a mixture of AAV-*Fgf13* plasmid and AAV-Mito-dsRed, anaesthetised mice were injected with 1.0 μL AAV at the following coordinates relative to bregma: AP, −3.0 mm; ML, ±1.3 mm; DV, −4.5 mm. Three weeks post injection, mice were subjected to subsequent drug treatment and preparation of the experimental model.

**1.2 Primary cell cultures and cell treatment**

In the establishment of primary neuron cultures, brain was isolated from embryos (embryonic day 15-16) of C57BL/6J mouse strain. Neurons were cultured for 7 days, and the medium was replaced with fresh medium every 3.5 days. In the establishment of primary glia cultures, the brain tissues of neonatal mice aged 1-3 day were utilized. The cell medium was replaced with fresh medium 24 h later and then refreshed every 3 days.

For the treatment of primary neurons, the cells were directly stimulated with different doses of MPP^+^ for vary time periods. Alternatively, the cells were first transfected with lentivirus (LV) packaged with *Fgf13* plasmid for three days or pre-treated with recombined FGF13 protein (Novoprotein; 10 ng·mL^-1^) for 1 h, and then treated with PD neurotoxins to establish an *in vitro* PD-like neuronal damage model. Specifically, the primary neurons were stimulated with 10 μM MPP^+^ for 24 h or with 1 μg·mL^-1^ α-synuclein for 72 h. For the treatment of mixed glial cells, recombined FGF13 protein (Novoprotein; 10 ng·mL^-1^) was used to incubate the cells for 1 h, followed by 200 μM MPP^+^ stimulation for 24 h. Alternatively, the mixed glial cell cultures in each well were incubated with the free mitochondria that were isolated from the neuronal medium in the corresponding well for 24 h. For the treatment of neuron-glia co-culture system, neurons were first transfected with LV packaged with *Fgf13* plasmid or a mixture of LV packaged with *Fgf13* plasmid and LV packaged with *Mtch2* shRNA, and then co-cultured with glia followed by the treatment of MPP^+^ or α-synuclein. Otherwise, the neurons were primed with Abacavir (MedChemExpress, Cat#HY-17423; 1 μM, 1 h), followed by the establishment of neuron-glia co-culture system and treatment of MPP^+^. The primary neurons co-cultured with glia, 10 μM MPP^+^ was used to stimulate the co-culture system for 24 h, otherwise 1 μg·mL^-1^ α-synuclein was used for 48 h.

**1.3 Immunohistochemistry and immunocytochemistry**

For immunohistochemistry (IHC) of brain slices, the brain tissues were dehydrated with 20% and then 30% sucrose dissolved in phosphate-buffered saline (PBS) for three days respectively after post-fixation in 4% paraformaldehyde (PFA). Then the brains embedded in OCT were cut into 20 μm-thick slices on coronal plane. Brain slices were simply washed three times in PBS. All slices were subsequently blocked with 5% BSA and 0.3% Triton X-100 in PBS (5% BSA/PBST), and then incubated with primary antibody in 4°C overnight followed by incubation of the secondary antibody at room temperature for 1 h. In Diaminobenzidin (DAB)-dependent IHC analysis, brain sections are incubated with 3% hydrogen peroxide to quench the endogenous peroxidase activity before blocking with 5% BSA/PBST. After rinsing with PBS following horseradish peroxidase (HRP)-labeled secondary antibody incubation, the slices were visualized by the DAB (Boster, Cat#AR1002) reaction for 5 minutes. IHC images were visualized under the microscope (Olympus) with stereo Investigator software, which was used to count the number of positive cells. Fluorescently labeled sections were visualized with the confocal scanning laser microscope (Carl Zeiss). For immunocytochemical staining, primary cells were rinsed with PBS and then fixed with 4% PFA for 20 min. Cell slides were then followed the same procedures as immunohistochemistry of brain slices.

The primary antibodies used were as follows: mouse anti-FGF13 antibody (1:300, Invitrogen, Cat#MA5-27705), rabbit anti-MAP2 antibody (1:500, Proteintech, Cat#17490-1-AP), rabbit anti-TH antibody (1:800, Proteintech, Cat#25859-1-AP), rabbit anti-GFAP antibody (1:500, Cell Signaling Technology, Cat#80778), rabbit anti-Iba-1 antibody (1:500, Asis biofarm, Cat#OB-PRB029) and rabbit anti-Neun antibody (1:400, Abcam, Cat#ab177487). The fluorescent secondary antibodies were as follows: Alexa Fluor™ 488 Goat anti-Rabbit IgG antibody (1:1000, Invitrogen, Cat#A32731), Alexa Fluor™ 555 Goat anti-Rabbit IgG antibody (1:1000, Invitrogen, Cat#A21428), Alexa Fluor™ 647 Goat anti-Rabbit IgG antibody (1:1000, Invitrogen, Cat#A32731), Alexa Fluor™ 488 Goat anti-Mouse IgG antibody (1:1000, Invitrogen, Cat#A11001), Alexa Fluor™ 555 Goat anti-Mouse IgG antibody (1:1000, Invitrogen, Cat#A21422).

**1.4 Nissl staining**

For Nissl staining, the brain slices were mounted onto the slides to dry naturally. The slides were soaked in the mixture of solution A and B from nissl staining kit (KeyGEN, Cat#KGA4104) as the manufacture's instruction, and then dehydrated with alcohol and xylene. The images were visualized under the microscope (Olympus), and the stereo Investigator software was used to count the number of positive cells.

**1.5 Protein extraction and immunoblots**

Midbrain tissues were lysed in lysis buffer containing protease inhibitor (Thermo Fisher, Cat#A32961). After centrifugation, protein concentration was measured and equal amount of protein was separated by sodium dodecyl sulphate-polyacrylamide gel electrophoresis (SDS-PAGE) electrophoresis. Proteins were then transferred from gel to 0.20 μm nitrocellulose membrane (Pall Corporation, Cat#66485). Membranes were blocked with 5% non-fat milk in Tris-buffered saline with Tween-20 (TBST) for 1 h, following by incubation with primary antibodies at 4°C overnight. Rabbit anti-TH antibody (1:1000, Proteintech, Cat#25859-1-AP), mouse anti-FGF13 antibody (1:1000, Invitrogen, Cat#MA5-27705), was used and rabbit secondary antibody was used at dilution of 1:5000. The bands were detected by ImageQuant LAS 4000 imaging system (GE Healthcare).

**1.6 Cell viability assay**

The primary neurons were seeded in 96 well plates with a density of 40,000 cells/well. After cell treatment, the culture medium was removed, and the suspension of 90 μl fresh culture medium and 10 μl Cell Counting Kit-8 solution (MedChemExpress, Cat#HY-K0301) was added into each well. Cell viability was detected by absorbance at 450 nm.

**1.7 Flow cytometry assay**

For flow cytometric analysis of live cells, primary neurons were stained with 1 μM Mitotracker green (Invitrogen, Cat#M46750) solution at 37°C in the dark for 30 min, 5 μM MitoSOX fluorescent dye (Invitrogen, Cat#M36008) at 37°C in the dark for 15 min, 10 μg·mL^-1^ JC-1 (Invitrogen, Cat#T3168) solution at 37°C in the dark for 30 min. The cells were then rinsed twice with PBS and then resuspended with cold PBS containing 1% FBS for flow cytometric analysis (FACS Calibur, BD, USA). For flow cytometric analysis of isolated mitochondria, cell medium of primary neurons was differentially centrifugated to isolate extracellular mitochondria and then stained with fluorescent dyes. The isolated mitochondria were then analyzed by flow cytometry (FACS Calibur, BD, USA). At least 5000 events per sample were collected for data analysis.

**1.8** **Isolation of extracellular mitochondria**

For the isolation of extracellular mitochondria, the supernatant obtained from primary neuron cultures was first subjected to centrifugation at 1,000 g for 10 minutes to pellet cellular debris. Subsequently, the resulting supernatant was centrifuged at 13,000 g for 30 minutes to collect the pellet that contained mitochondria. To detect mass and quality of isolated mitochondria, the mitochondrial fluorescent probes including Mitotracker green (Invitrogen, Cat#M46750), MitoSOX Red (Invitrogen, Cat#M36008) and JC-1 (Invitrogen, Cat#T3168) were used. Additionally, transmission electron microscopy was applied for a more detailed morphological observation.

**1.9 Flow cytometry sorting of extracellular mitochondria**

To purify extracellularly secreted mitochondria via flow cytometry sorting, primary neurons were transfected with Mito-dsRed lentivirus to specifically label neuronal mitochondria. Subsequently, mitochondria present in the culture medium of primary neurons were isolated and purified using flow cytometry sorting. The purified mitochondria were then used to stimulate primary microglia, primary astrocytes, or mixed glial cells for 24 h, after which the cells were subjected to further experimental procedures.

**1.10 Statistics**

Statistical significance was determined using GraphPad Prism 8. Unpaired two-tailed Student’s t-test, one-way ANOVA, two-way ANOVA or pearson correlation test was conducted according to test requirements. **p* < 0.05, ***p* < 0.01, and ****p* < 0.001 were considered significant.

1. **Supplementary Figures and Legends**

**2.1 Figure S1:
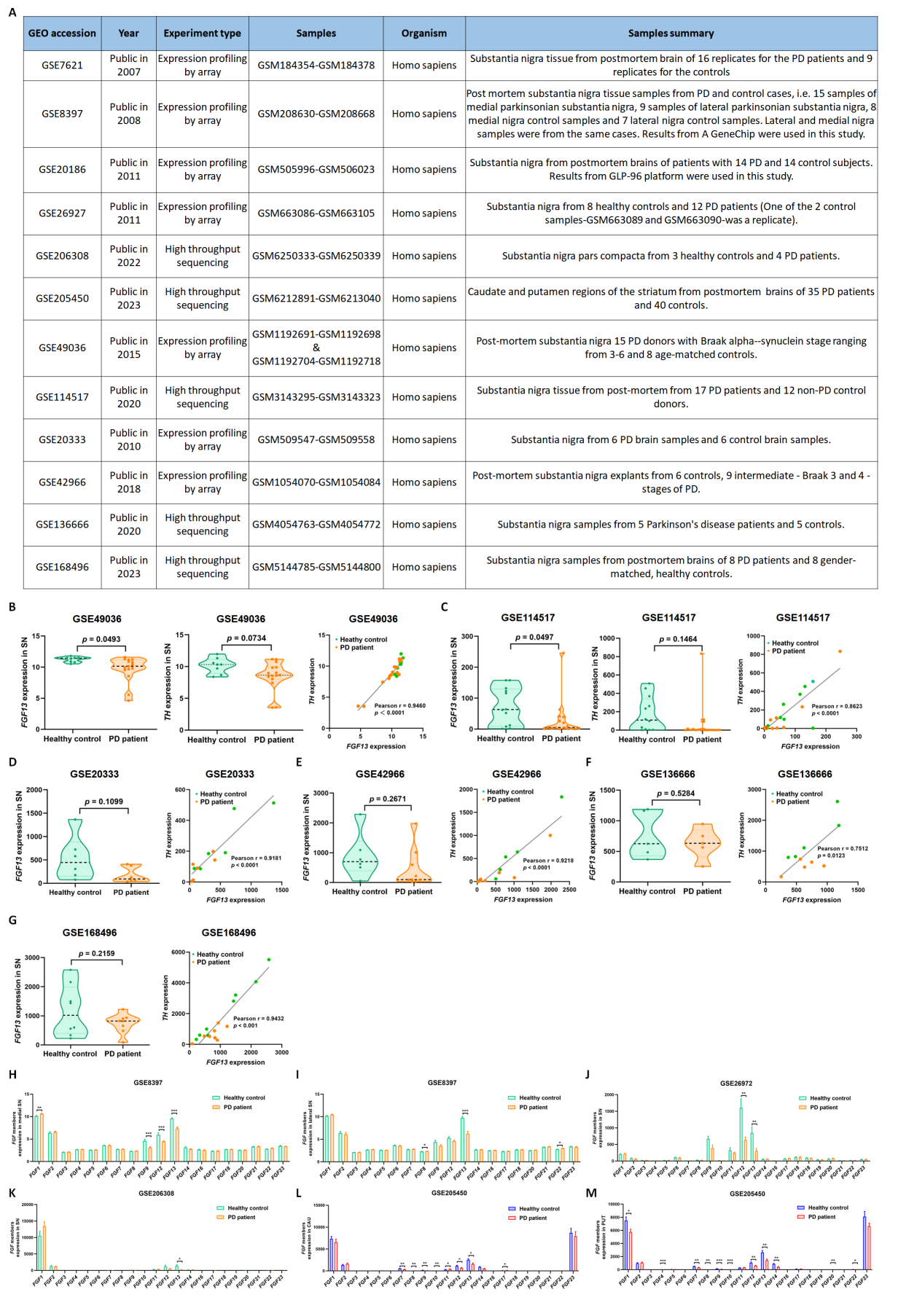
**

**Figure S1. Information of the publicly GEO datasets used in this study, *Fgf13* and *Th* levels in the SN of PD patients. Related to Figure 1.**

1. Information of publicly GEO datasets based on tissues from PD patients. **B.** *FGF13* and *TH* gene expression in the SN of PD patients (GSE49036: n = 8 for the healthy group, n = 15 for the disease group); and correlation of *Fgf13* and *Th* gene expression. **C.** *FGF13* and *TH* gene expression in the SN of PD patients (GSE114517: n = 12 for the healthy group, n = 17 for the disease group); and correlation of *Fgf13* and *Th* gene expression. **D.** *FGF13* gene expression in the SN of PD patients (GSE20333: n = 6 for the healthy group, n = 6 for the disease group); and correlation of *FGF13* and *TH* gene expression. **E.** *FGF13* gene expression in the SN of PD patients (GSE42966: n = 6 for the healthy group, n = 9 for the disease group); and correlation of *FGF13* and *TH* gene expression. **F.** *FGF13* gene expression in the SN of PD patients (GSE136666: n = 5 for the healthy group, n = 5 for the disease group); and correlation of *FGF13* and *TH* gene expression. **G.** *FGF13* gene expression in the SN of PD patients (GSE168496: n = 8 for the healthy group, n = 8 for the disease group); and correlation of *FGF13* and *TH* gene expression. **H-I.** Gene expression of all FGF family members in the medial and lateral SN of PD patients from the GSE8397-GPL96 platform. **J.** Gene expression of all FGF family members in the SN of PD patients from GSE29672. **K.** Gene expression of all FGF family members in the SN of PD patients from GSE206308. **L-M.** Gene expression of all FGF family members in the caudate (CAU) and putamen (PUT) of the PD patients from GSE205450. All data are presented as the mean ± s.e.m. In **B-G**, an unpaired two-tailed Student’s t-test and a pearson correlation test were used. In **H-M**, an unpaired two-tailed Student’s t-test.

**2.2 Figure S2：
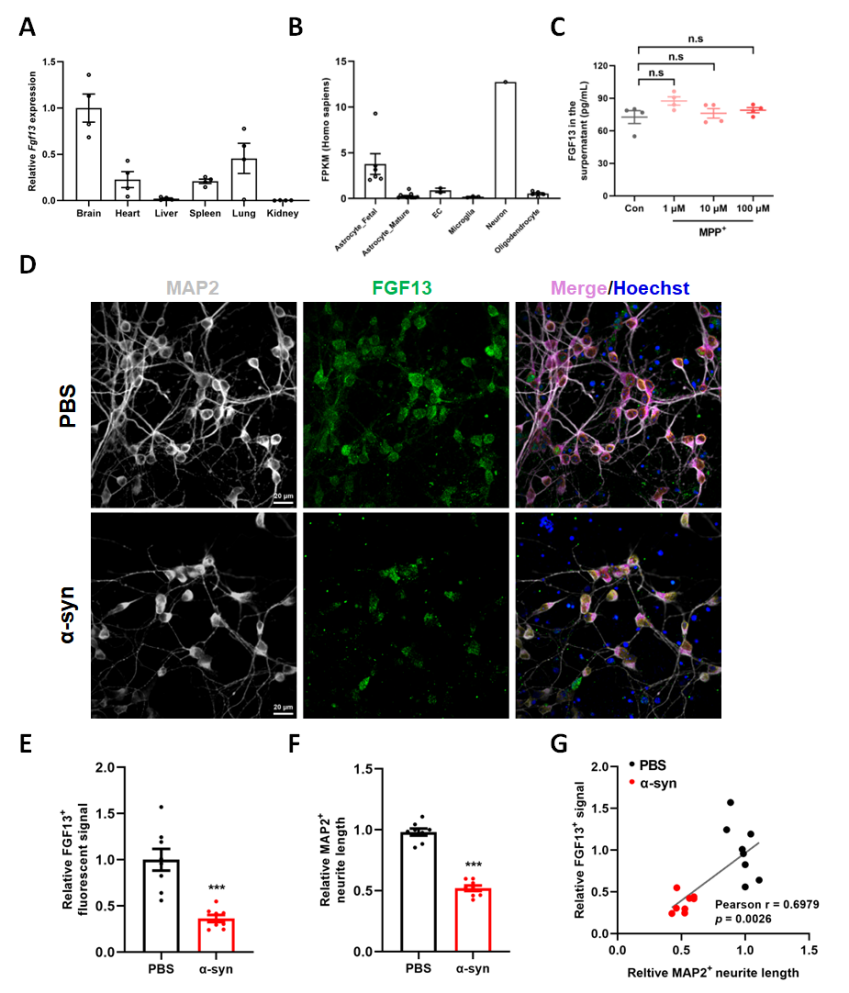
**

**Figure S2. *Fgf13* expression in various mouse tissues and in primary neurons with α-synuclein stimulation. Related to Figure 2.**

1. The expression of *Fgf13* in different organs of mice (n = 4 mice per group). **B.** *Fgf13* gene expression in different cell types of human brain from a published RNA-seq dataset (GSE73721: n = 6 for Astrocyte_Fetal, n = 12 for Astrocyte_mature, n = 2 for EC, n = 2 for Microglia, n = 1 for neuron, n = 5 for Oligodendrocyte; EC means endothelial cell). **C.** FGF13 levels in cell medium of primary neurons with MPP^+^ stimulation (n = 4 replicate experiments per group). **D.** Representative fluorescent images of MAP2 (grey) and FGF13 (green) in primary neurons stimulated with α-synuclein. **E.** Quantitative analysis of FGF13^+^ signal in primary neurons (8 images per group from 4 independent experiments). **F.** Quantitative analysis of MAP2^+^ neurite length in primary neurons (8 images per group from 4 independent experiments). **G.** Correlation of relative FGF13^+^ signals and relative MAP2^+^ neurite length in primary neurons stimulated with α-synuclein. All data are presented as the mean ± s.e.m. ****p* < 0.001 *vs.* PBS group. In **C**, a one-way ANOVA with Dunnett’s multiple comparisons test was used. In **E-F**, an unpaired two-tailed Student’s t-test was used. In **G**, a pearson correlation test was used.

**2.3 Figure S3：**


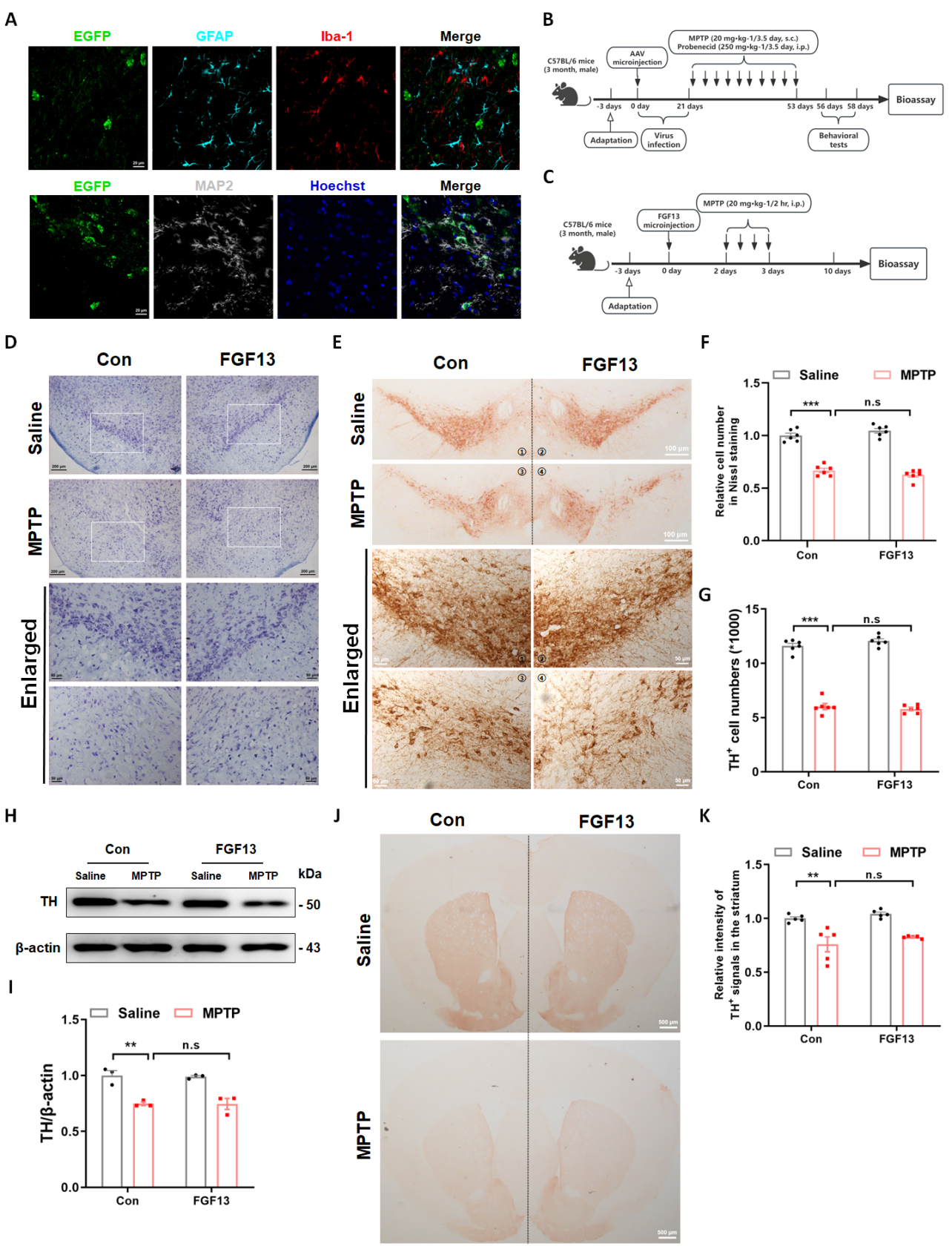
**Figure S3. FGF13 supplementation shows no effect on loss of nigrostriatal neurons. Related to Figure 3.**

1. Representative fluorescent images illustrating the colocalization of EGFP with GFAP (turquoise), Iba-1 (red), and MAP2 (grey) in brain slices. **B.** Schematic illustration of the experimental design for the chronic MPTP-induced PD-like model in mice microinjected with AAV-*Fgf13*. **C.** Schematic illustration of the experimental design for the acute MPTP-induced PD-like model in mice microinjected with recombinant FGF13 protein. **D.** Representative images of Nissl staining in the SNc. **E.** Representative immunohistological images of TH in the SNc. **F.** Stereological counts of TH-positive cells in the SNc (n = 6 mice per group). **G.** Representative immunoblots of TH in the SNc. **H.** Quantitative analysis of TH proteins in immunoblotting (n = 3 mice per group). **G.** Representative TH-IHC in the striatum of mice. **I.** Relative optical intensity of TH in the striatum of mice (n = 5 mice per group). All data are presented as the mean ± s.e.m. ***p* < 0.01 and ****p* < 0.001 *vs.* Con Saline group; n.s means no significance. Statistical comparison was performed using two-way ANOVA.

**2.4 Figure S4：**

**
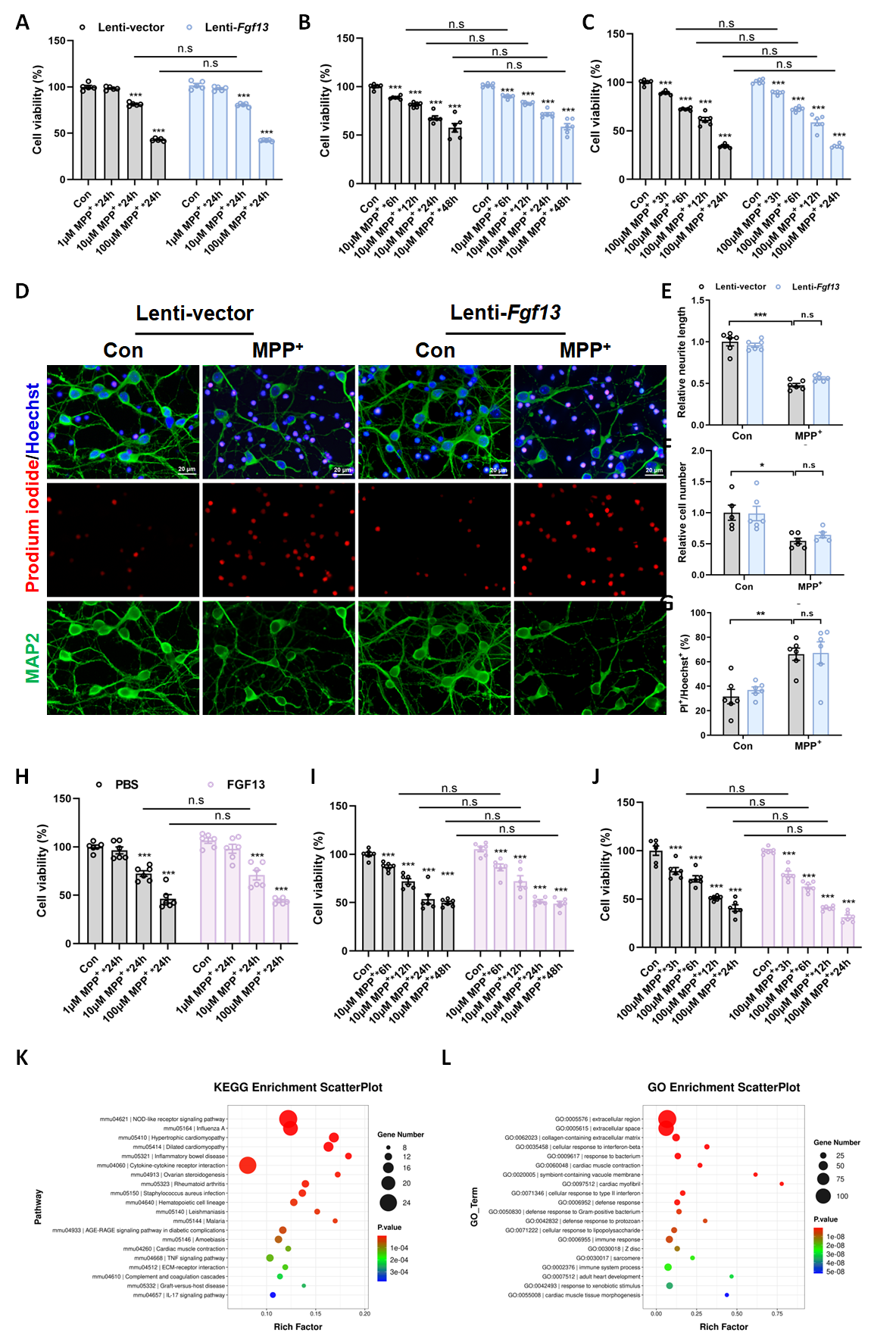
**

**Figure S4. *Fgf13* over-expression and recombinant** **FGF13 protein show no effects on neuronal damage under PD toxin in primary neuron cultures, but mitigates inflammatory pathways in the nigrostriatal tissues of mice. Related to Figure 4.**

1. **C.** Cell viability of primary neurons transfected with Lenti-*Fgf13*, followed by the treatment of MPP^+^ treatment (5-6 replicates per group). **D.** Representative fluorescent images of MAP2 (green), prodium iodide (PI for labeling the nucleus of damaged cell, red) and Hoechst in primary neurons stimulated with 10 μM MPP^+^ for 24 h. **E-F.** Relative neurite length and cell number in MAP2^+^ neurons (5-6 images from 3 independent experiments). **G.** Percent of damaged cell among all cells (6 images from 3 independent experiments). **H-J.** Cell viability of primary neurons with pre-incubation of recombinant FGF13 protein, followed by the treatment of MPP^+^ treatment (6 replicates per group). **K-L.** KEGG pathway and GO enrichment analysis of differential pathways. All data are presented as the mean ± s.e.m. **p* < 0.05, ***p* < 0.01 and ****p* < 0.001 *vs.* Lenti-vector Con group or PBS Con; n.s means no significance. Statistical comparison was performed using two-way ANOVA.

**2.5 Figure S5：
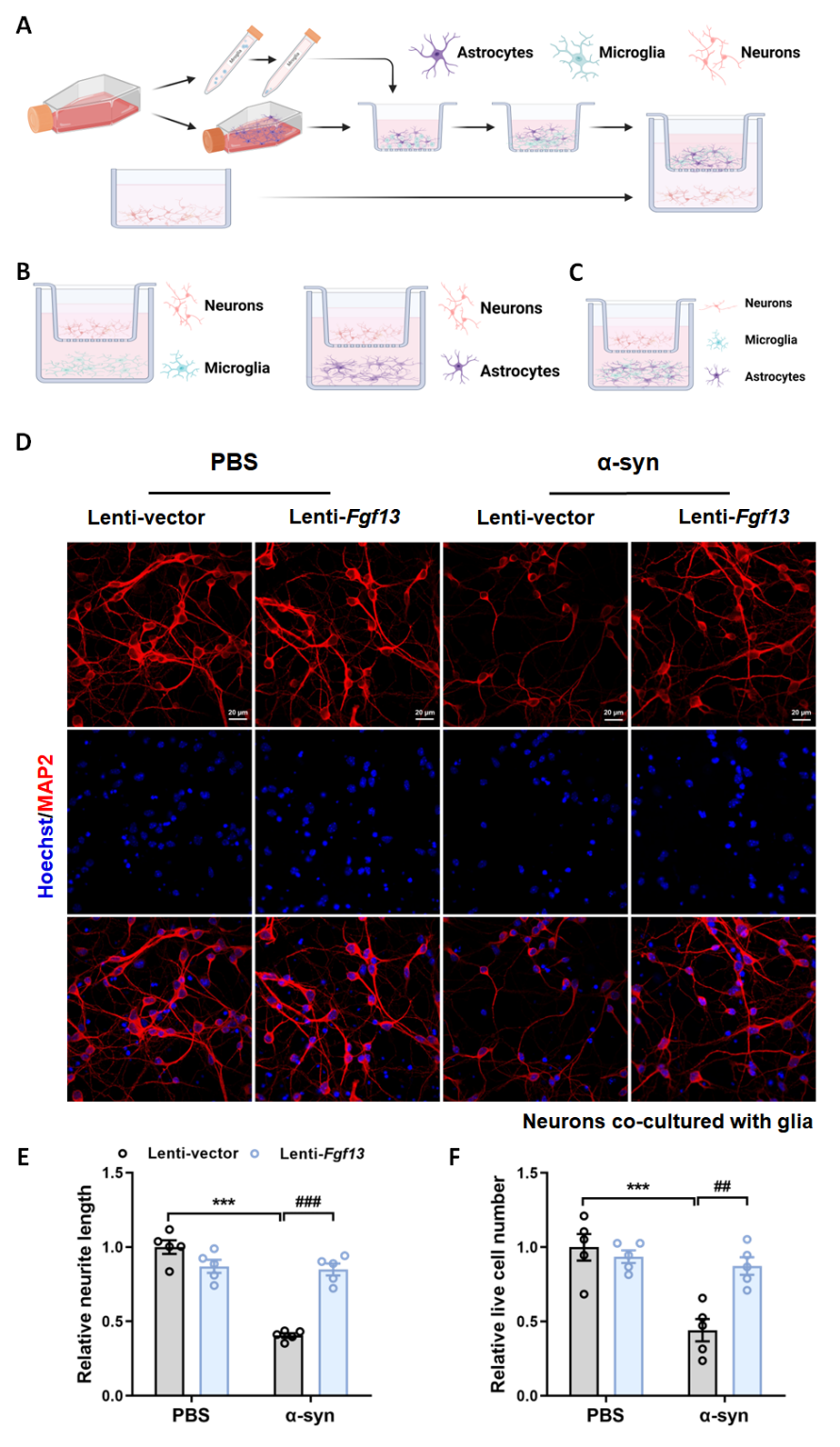
**

**Figure S5. *Fgf13* over-expression mitigates α-syn-induced neuronal damage in neuron-glia co-culture system. Related to Figure 5.**

**A-C.** Schematic diagrams of the neuron-glia co-culture system using transwell chambers. **A** corresponds to **Figure 5A-F** and **5L-Q**; **B** corresponds to **Figure 5G-K**; **C** corresponds to **Figure 5R**. **D.** Representative fluorescent images of MAP2 (red) and Hoechst in primary neurons stimulated with α-synuclein. **E-F.** Relative neurite length and cell number in MAP2^+^ neurons with α-synuclein treatment (5 images from 3 independent experiments). All data are presented as the mean ± s.e.m. ****p* < 0.001 *vs.* Lenti-vector PBS group; ##*p* < 0.01 and ###*p* < 0.001 *vs.* Lenti-vector α-syn group. Statistical comparison was performed using two-way ANOVA.

**2.6 Figure S6：**
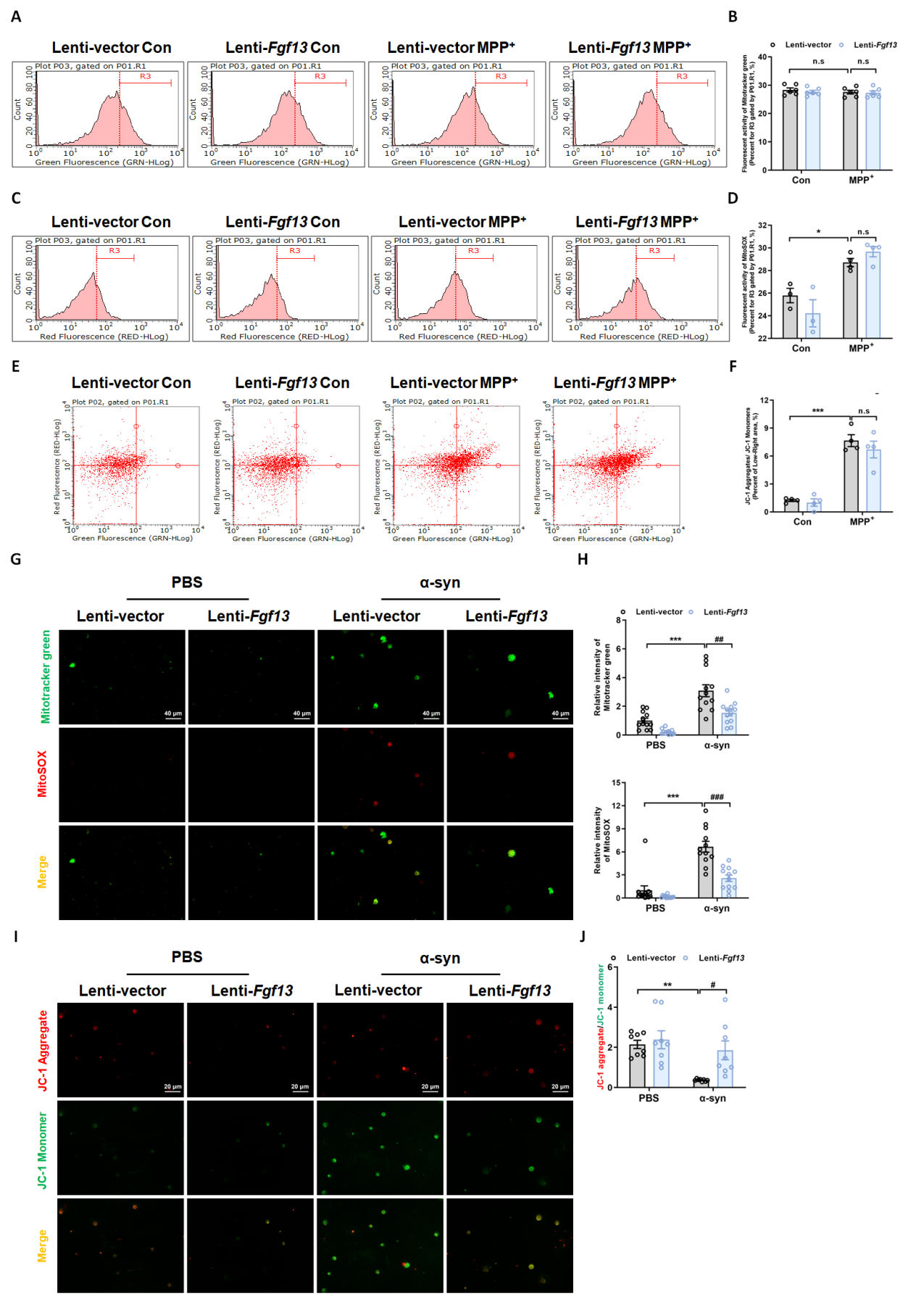


**Figure S6. *Fgf13* over-expression shows no effects on mitochondrial mass and functions in primary neurons under PD toxin, but mitigates malfunctions of extracellular mitochondria in primary neurons under PD toxin. Related to Figure 6.**

1. Mitotracker green signals in primary neurons were analyzed by flow cytometry. **B.** Quantitative analysis of Mitotracker green signals in primary neurons (n = 6 replicates per group). **C.** MitoSOX signals in primary neurons were analyzed by flow cytometry. **D.** Quantitative analysis of MitoSOX Red signals in primary neurons (n = 4 replicates per group). **E.** Ratio of JC-1 aggregate to JC-1 monomer in primary neurons were analyzed by flow cytometry. **F.** Quantitative analysis of JC-1 aggregate/JC-1 monomer in primary neurons (n = 4 replicates per group). **G.** Representative fluorescent images of extracellular mitochondria stained with Mitotracker green and MitoSOX Red. **H.** Quantitative analysis of Mitotracker green signals and MitoSOX Red signals (12 images from 3 independent experiments). **I.** Representative fluorescent images of extracellular mitochondria stained with JC-1. **J.** Ratio of JC-1 aggregate to JC-1 monomer in extracellular mitochondria (8 images from 4 independent experiments). All data are presented as the mean ± s.e.m. **p* < 0.05, ***p* < 0.01 and ****p* < 0.001 *vs.* Vector Con group or Lenti-vector PBS group; #*p* < 0.05, ##*p* < 0.01 and ###*p* < 0.01 *vs.* Lenti-vector α-syn group; n.s means no significance. Statistical comparison was performed using two-way ANOVA.

**2.7 Figure S7：**

**
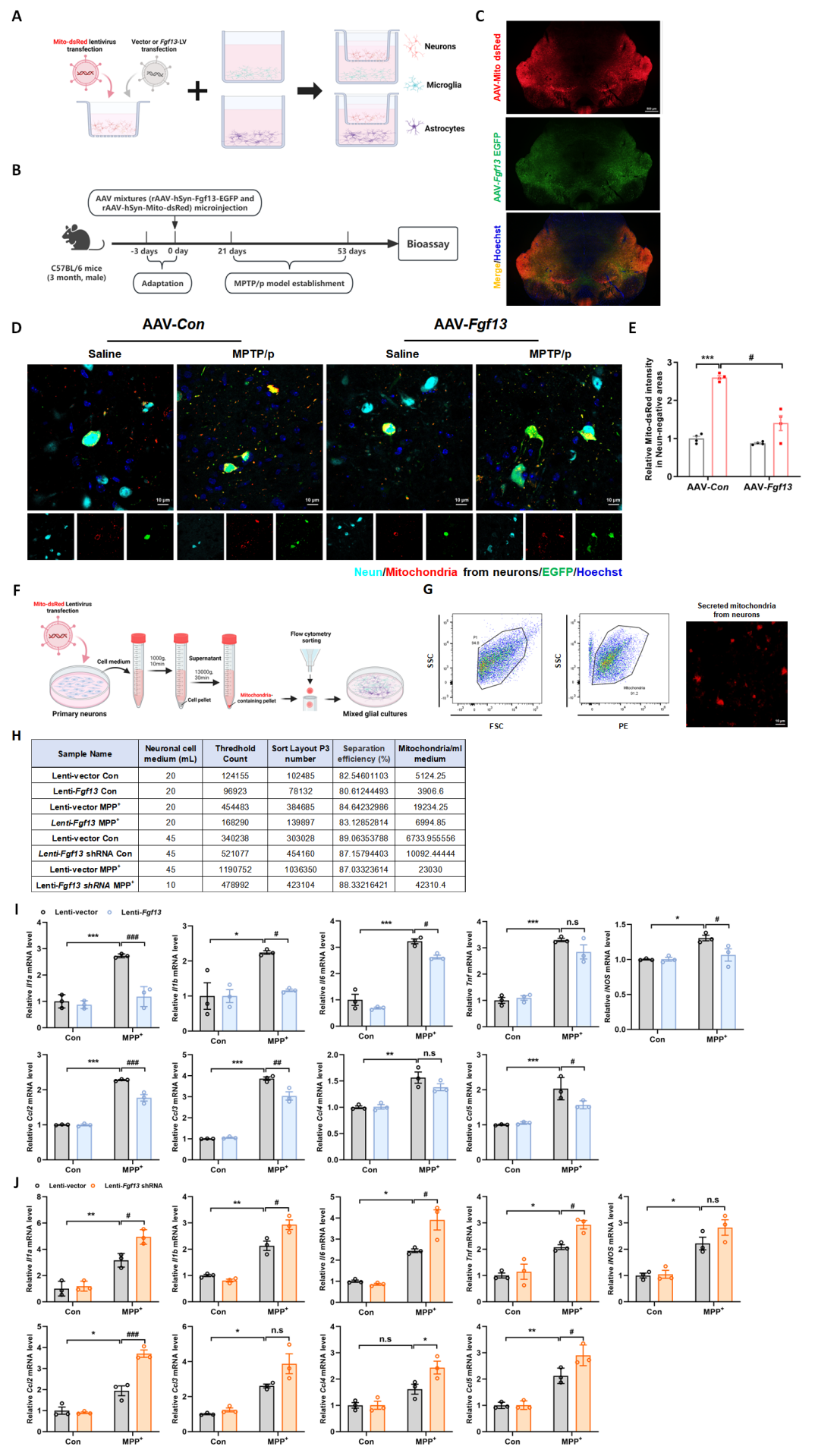
Figure S7. Overexpression of *Fgf13* inhibits mitochondrial efflux from neurons of *Fgf13* over-expression on mitochondrial release. Related to Figure 7.**

1. Schematic illustration of the neuron-glia co-culture system utilizing transwell chambers. **B.** Schematic illustration of the experimental design for the MPTP/p-induced PD-like model in mice microinjected with a mixture of AAV-*Fgf13* and AAV-Mito-dsRed. **C.** The colocalization of EGFP and Mito-dsRed in the midbrain after AAV injection. **D.** Representative fluorescent images illustrating EGFP and Mito-dsRed with Neun (neuronal marker, turquoise) in brain slices. **E.** Relative Mito-dsRed fluorescent intensity in non-neuronal area. **F.** Schematic representation of the experiment for purifying extracellular mitochondria from neurons labeled with Mito-dsRed using flow cytometric sorting. **G.** The strategy for purifying extracellular mitochondria from neurons using flow cytometric sorting, along with representative images of the purified mitochondria. **H.** The approximate quantity of sorted mitochondria in each sample using flow cytometric sorting. **I-J**. The levels of pro-inflammatory and chemokine genes in mixed glial cells treated with the purified mitochondria (n = 3 replicates per group). All data are presented as the mean ± s.e.m. **p* < 0.05, **p* < 0.01 and ****p* < 0.001 *vs.* Lenti-vector Con or AAV-*Con* Saline group; #*p* < 0.05, ##*p* < 0.01 and ###*p* < 0.001 *vs.* Lenti-vector MPP^+^ or AAV-*Con* MPTP/p group; n.s means no significance. Statistical comparison was performed using two-way ANOVA.

**2.8 Figure S8：
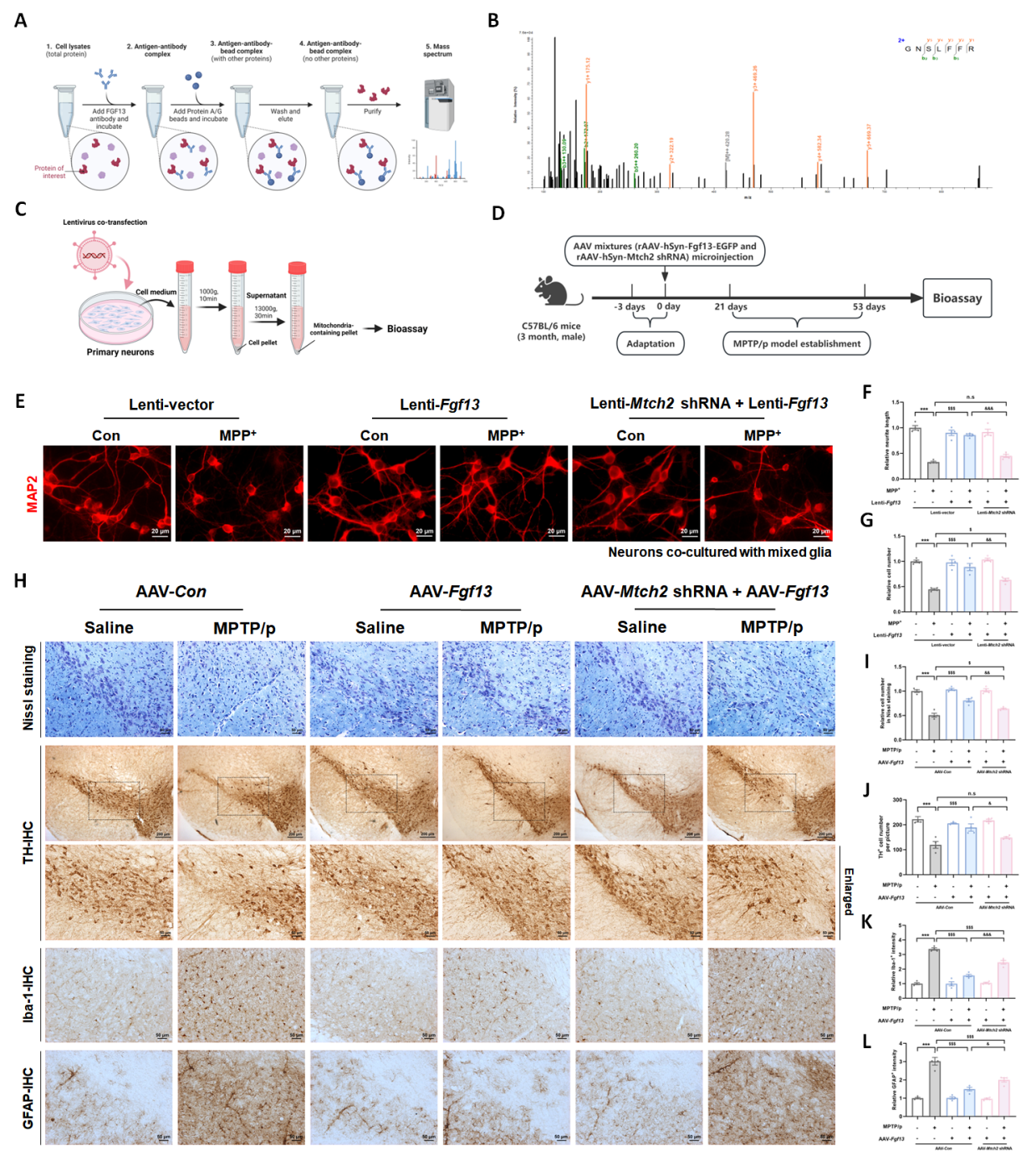
**

**Figure S8. *Mtch2* knockdown partially cancels the effects of *Fgf13* over-expression on mitochondrial release. Related to Figure 8.**

1. Flow chart of FGF13-binding proteins in primary neurons by mass spectrum. **B.** Mass spectrum of MTCH2 in FGF13-immunopricipitated proteins in primary neurons. **C**. Experimental schematic for collecting extracellular mitochondria from neurons co-transfected with LV carrying the *Fgf13* plasmid and LV carrying *Mtch2* shRNA. **D.** Schematic illustration of the experimental design for the MPTP/p-induced PD-like model in mice microinjected with a mixture of AAV-*Fgf13* and AAV-*Mtch2* shRNA. **E.** Representative fluorescent images of MAP2 (red) in primary neurons co-cultured with the mixed glia. **F-G.** Relative neurite length and cell number in MAP2-positive neurons with α-synuclein treatment (4 independent images). **H.** Representative images depicting Nissl staining, TH immunohistochemistry, as well as GFAP and Iba-1 immunohistochemistry within the SNc. **I.** Relative cell number in the Nissl staining. **J.** Relative number of TH-positive cells in the TH immunohistochemistry. **K.** Relative intensity of Iba-1-positive staining in immunohistochemistry. **L.** Relative intensity of GFAP-positive staining in immunohistochemistry. All data are presented as the mean ± s.e.m. ****p* < 0.001 *vs.* Lenti-vector Con or AAV-*Con* Saline group; $*p* < 0.05 and $$$*p* < 0.001 *vs.* Lenti-vector MPP^+^ or AAV-Con MPTP/p group; &*p* < 0.05, &&*p* < 0.01 and &&&*p* < 0.001 *vs.* Lenti-*Fgf13* MPP^+^ or AAV-*Fgf13* MPTP/p group; n.s means no significance. Statistical comparison was performed using one-way ANOVA with Dunnett’s multiple comparisons test.

**2.9 Figure S9：
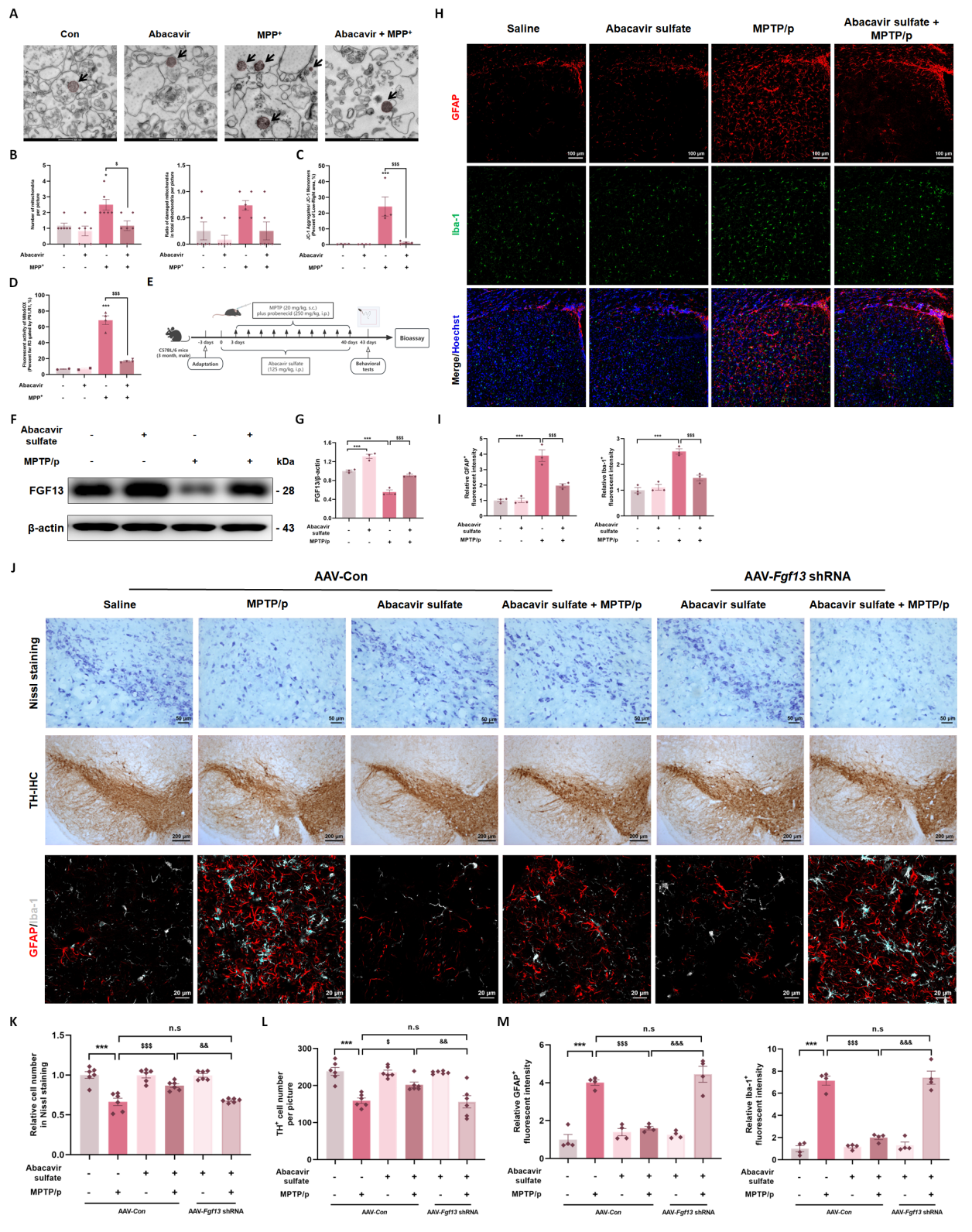
**

**Figure S9. Abacavir reduces malfunctions of extracellular mitochondria in primary neurons under PD stress, and inhibits glial activation in the striatum of PD-like mice. These effects were dependent on FGF13. Related to Figure 9.**

1. TEM images of extracellular mitochondria (pink circles). **B.** Number of extracellular mitochondria and percentage of damaged mitochondria among all mitochondria per picture (6 pictures from 3 replicates per group). **C.** Quantitative analysis of JC-1 aggregate/JC-1 monomer in extracellular mitochondria by flow cytometry (n = 4 replicates per group). **D.** Quantitative analysis of MitoSOX Red signals in flow cytometry (n = 2-4 replicates per group). **E.** Experimental schematic for Abacavir sulfate administration in MPTP/p-induced PD-like mice. **F-G.** Representative immunoblots of FGF13 in primary neurons and the quantitative analysis (n = 3 replicate experiments per group). **H.** Representative fluorescent images of GFAP (green) and Iba-1 (red) in the striatum of mice. **I.** Quantitative analysis of GFAP^+^ and Iba-1^+^ fluorescent signals in the striatum (n = 3 mice per group). **J.** Representative images of Nissl staining, TH immunohistological staining; and representative fluorescent images of GFAP (red) and Iba-1 (grey) in the SN. **K.** Relative neuron numbers in the SN by Nissl staining (n = 6 mice per group). **L.** Relative dopaminergic neuron numbers in the SN by TH-IHC staining (n = 6 mice per group). **M.** Quantitative analysis of GFAP+ and Iba-1+ fluorescent intensity in the SN (n = 4 mice per group). All data are presented as the mean ± s.e.m. **p* < 0.05 and ****p* < 0.001 *vs.* Con or Saline group; $*p* < 0.05 and $$$*p* < 0.001 *vs.* MPP^+^ or MPTP/p or AAV-Con MPTP/p group; &&*p* < 0.01 and &&&*p* < 0.001 *vs.* AAV-*Fgf13* shRNA MPTP/p group; n.s means no significance. Statistical comparison was performed using one-way ANOVA with Dunnett’s multiple comparisons test.

**Supplementary Table 1**

Primers used for qRT-PCR.

**Supplementary Table 2**

Primers for plasmids of shRNA construction.

**Supplementary Table 1.** Primers used for qRT-PCR

| **Primers** | **Sequence (5' to 3')** |
| --- | --- |
| *Fgf13*-Forward | TCTTCGGGTGGTGGCTATTC |
| *Fgf13*-Reverse | ATCGGGAGAACTCCGTGAG |
| *Il1a*-Forward | CGAAGACTACAGTTCTGCCATT |
| *Il1a*-Reverse | GACGTTTCAGAGGTTCTCAGAG |
| *Il1b*-Forward | TCAGGCAGGCAGTATCACTC |
| *Il1b*-Reverse | CATGAGTCACAGAGGATGGG |
| *Il6*-Forward | ATCCAGTTGCCTTCTTGGGACTGA |
| *Il6*-Reverse | TAAGCCTCCGACTTGTGAAGTGGT |
| *Il12b*-Forward | TGGTTTGCCATCGTTTTGCTG |
| *Il12b*-Reverse | ACAGGTGAGGTTCACTGTTTCT |
| *Tnf*-Forward | TTGCTCTGTGAAGGGAATGG |
| *Tnf*-Reverse | GGCTCTGAGGAGTAGACAATAAAG |
| *iNOS*-Forward | GAACGGAGAACGTTGGATTTG |
| *iNOS*-Reverse | TCAGGTCACTTTGGTAGGATTT |
| *Ccl2*-Forward | TTAAAAACCTGGATCGGAACCAA |
| *Ccl2*-Reverse | GCATTAGCTTCAGATTTACGGGT |
| *Ccl3*-Forward | TTCTCTGTACCATGACACTCTGC |
| *Ccl3*-Reverse | CGTGGAATCTTCCGGCTGTAG |
| *Ccl4*-Forward | TTCCTGCTGTTTCTCTTACACCT |
| *Ccl4*-Reverse | CTGTCTGCCTCTTTTGGTCAG |
| *Ccl5*-Forward | GCTGCTTTGCCTACCTCTCC |
| *Ccl5*-Reverse | TCGAGTGACAAACACGACTGC |
| *Arg1*-Forward | CTCCAAGCCAAAGTCCTTAGAG |
| *Arg1*-Reverse | AGGAGCTGTCATTAGGGACATC |
| *Mrc1*-Forward | CTCTGTTCAGCTATTGGACGC |
| *Mrc1*-Reverse | CGGAATTTCTGGGATTCAGCTTC |
| *Nlrp3*-Forward | ATTACCCGCCCGAGAAAGG |
| *Nlrp3*-Reverse | TCGCAGCAAAGATCCACACAG |
| *Cxcl3*-Forward | ATCCCAACGGTGTCTGGATG |
| *Cxcl3*-Reverse | GCAAGTAGATGCAATTATACCCGT |
| *Gadph*-Forward | AACGACCCCTTCATTGAC |
| *Gadph*-Reverse | TCCACGACATACTCAGCAC |

**Supplementary Table 2.** Primers for plasmids of shRNA construction

| **Names** | **Oligonucleotides** |
| --- | --- |
| Negative control shRNA | 5’-CACCGTTCTCCGAACGTGTCACGTCAAGAGATTACGTGACACGTTCGGAGAATTTTTTG-3’ |
|  | 5’-GATCCAAAAAATTCTCCGAACGTGTCACGTAATCTCTTGACGTGACACGTTCGGAGAAC-3’ |
| *Fgf13* shRNA | 5’-CACCGCACTTACACTCTGTTTAACCTTCAAGAGAGGTTAAACAGAGTGTAAGTGCTTTTTTG-3’ |
|  | 5’-GATCCAAAAAAGCACTTACACTCTGTTTAACCTCTCTTGAAGGTTAAACAGAGTGTAAGTGC-3’ |
| Negative control shRNA | 5’-CCGGCAACAAGATGAAGAGCACCAACTCGAGTTGGTGCTCTTCATCTTGTTGTTTTTTG-3’ |
|  | 5’-CAAAAACACAAAGATGAAGAGCACCAACTCGAGTTGGTGCTCTTCATATTGTTGCCGG-3’ |
| *Mtch2* shRNA | 5'-CCGGAGCCGCTCATGTACGTGAAAGCTCGAGCTTTCACGTACATGAGCGGCTTTTTTG-3' |
|  | 5'-AATTCAAAAAAGCCGCTCATGTACGTGAAAGCTCGAGCTTTCACGTACATGAGCGGCT-3' |
